# Bimodal dynamics of primary metabolism-related responses in tolerant potato-*Potato virus Y* interaction

**DOI:** 10.1186/s12864-015-1925-2

**Published:** 2015-09-19

**Authors:** Tjaša Stare, Živa Ramšak, Andrej Blejec, Katja Stare, Neža Turnšek, Wolfram Weckwerth, Stefanie Wienkoop, Dominik Vodnik, Kristina Gruden

**Affiliations:** Department of Biotechnology and Systems Biology, National Institute of Biology, Vecna pot 111, Ljubljana, Slovenia; Department of Ecogenomics and Systems Biology, Faculty of Life Sciences, University of Vienna, Vienna, Austria; Department of Agronomy, Biotechnical Faculty, University of Ljubljana, Ljubljana, Slovenia

**Keywords:** Plant-pathogen interactions, *Potato virus Y*, Potyviridae, Salicylic acid, *Solanum tuberosum*, Whole transcriptome analysis, Shot-gun proteomics, Photosynthetic parameters

## Abstract

**Background:**

*Potato virus Y* (PVY) is a major pathogen that causes substantial economic losses in worldwide potato production. Different potato cultivars differ in resistance to PVY, from severe susceptibility, through tolerance, to complete resistance. The aim of this study was to better define the mechanisms underlying tolerant responses of potato to infection by the particularly aggressive PVY^NTN^ strain. We focused on the dynamics of the primary metabolism-related processes during PVY^NTN^ infection.

**Results:**

A comprehensive analysis of the dynamic changes in primary metabolism was performed, which included whole transcriptome analysis, nontargeted proteomics, and photosynthetic activity measurements in potato cv. Désirée and its transgenic counterpart depleted for accumulation of salicylic acid (NahG-Désirée). Faster multiplication of virus occurred in the NahG-Désirée, with these plants developing strong disease symptoms. We show that while the dynamics of responses at the transcriptional level are extensive and bimodal, this is only partially translated to the protein level, and to the final functional outcome. Photosynthesis-related genes are transiently induced before viral multiplication is detected and it is down-regulated later on. This is reflected as a deficiency of the photosynthetic apparatus at the onset of viral multiplication only. Interestingly, specific and constant up-regulation of some RuBisCO transcripts was detected in Désirée plants, which might be important, as these proteins have been shown to interact with viral proteins.

In SA-deficient and more sensitive NahG-Désirée plants, consistent down-regulation of photosynthesis-related genes was detected. A constant reduction in the photochemical efficiency from the onset of viral multiplication was identified; in nontransgenic plants this decrease was only transient. The transient reduction in net photosynthetic rate occurred in both genotypes with the same timing, and coincided with changes in stomatal conductivity.

**Conclusions:**

Down-regulation of photosynthesis-related gene expression and decreased photosynthetic activity is in line with other studies that have reported the effects of biotic stress on photosynthesis. Here, we additionally detected induction of light-reaction components in the early stages of PVY^NTN^ infection of tolerant interaction. As some of these components have already been shown to interact with viral proteins, their overproduction might contribute to the absence of symptoms in cv. Désirée.

**Electronic supplementary material:**

The online version of this article (doi:10.1186/s12864-015-1925-2) contains supplementary material, which is available to authorized users.

## Background

Potato (*Solanum tuberosum* L.) is the most widely grown tuber crop in the world, and the fourth largest food crop in terms of fresh produce, after rice, wheat and tomato. *Potato virus Y *(PVY) is a member of the *Potyviridae* family, and economically, it is one of the most important potato pathogens, with a worldwide spread [1]. Several strains of PVY have been isolated that differ at the molecular and biological levels. PVY^NTN^ is an aggressive isolate that induces severe symptoms in sensitive potato cultivars, with the development of potato tuber necrotic ringspot disease, thus resulting in major economic losses [2, 3]. Different potato cultivars show different levels of sensitivity to this particular viral strain, from susceptibility, through tolerance, to complete resistance (reviewed in [2]).

Plant defenses against pathogens are regulated at the molecular level by a network of interconnecting signal transduction pathways, of which salicylic acid (SA) is an important component [4, 5]. SA has been shown to mediate resistance in many compatible plant-virus interactions and its deficiency leads to an impairment of the defense responses and susceptibility to pathogen attack [6, 7]. Depending upon the virus and the host, SA can induce inhibition of viral replication, or cell-to-cell or long distance viral movement [4, 8]. In recent years, it has become clear that plant defense responses are complex, and that they arise from crosstalk between different hormonal signaling pathways that enable specificity of responses to different pathogens and fine-tuning of defense responses [5, 9].

Pathogens that attack plants promote massive reprogramming of the plant metabolism for the synthesis of chemical defenses—a process that can be costly in terms of plant growth and fitness [10]. Plants must balance potentially competing demands for resources, to support both their defense and their requirements for cellular maintenance, growth and reproduction. Previous studies have shown that lowered plant growth rates in virus-infected plants can be attributed mainly to impaired photosynthesis, albeit experimental data that relate biotic stress and photosynthesis are often inconsistent. On the one hand, a decline in the rate of photosynthesis following attack by insects or pathogens has been documented; and on the other hand, examples of compensatory stimulation of photosynthesis have also been reported (reviewed in [11]). Most of these studies have focused on different static time points after virus infection, with very few exceptions that have followed the dynamics of targeted gene expression [12, 13]. However, to understand the reprogramming of plant metabolism, it is not sufficient to look only at a single ‘screen shoot’ of the plant status. Instead, dynamic ranges of these responses should be monitored.

The potato-PVY interaction has been studied previously at both morphological and biochemical levels, as well as at the gene expression level (reviewed in [2]). Recently, we performed a time series analysis of responses in compatible potato-PVY^NTN^ interaction [13]. To determine the role of SA in this interaction, the NahG-Désirée transgenic line that expresses salicylate hydroxylase, which catalyzes the conversion of SA to catechol [7, 13, 14], was also analyzed. We have shown that SA depletion renders Désirée more susceptible to PVY^NTN^ infection. The virus was able to multiply faster, and strong disease symptoms appeared. We followed the dynamics of the expression of selected genes, and show that photosynthesis-related chlorophyll a, b binding protein 4 and ribulose 1,5-bisphosphate carboxylase/ oxygenase (RuBisCO) activase genes are strongly down-regulated in NahG-Désirée plants compared to their non-transgenic counterparts [13].

With this study, we aimed to better understand the disease development and the relationships between primary metabolism of potato and its defense response to infection with PVY^NTN^. A comprehensive analysis of the dynamic changes in primary metabolism of potato was performed, which included whole transcriptome analysis, nontargeted proteomics and photosynthetic activity measurements in the plants of cv. Désirée and the transgenic NahG-Désirée. We show that while the dynamics of responses at the transcriptional level are extensive and bimodal, this is only partially translated to the protein level, and to the final functional outcome. The activity of photosynthesis-related genes is transiently induced before viral multiplication is detected in the infected leaf, and it is down-regulated later on. This is reflected as a deficiency of the photosynthetic apparatus at the onset of viral multiplication only. In SA-deficient and more sensitive NahG-Désirée plants, however, the down-regulation of the photosynthetic apparatus is detected at all levels.

## Results and discussion

### The distribution of viral RNA between lamina and veins differs in symptomatic leaves

**Fig. 1:**
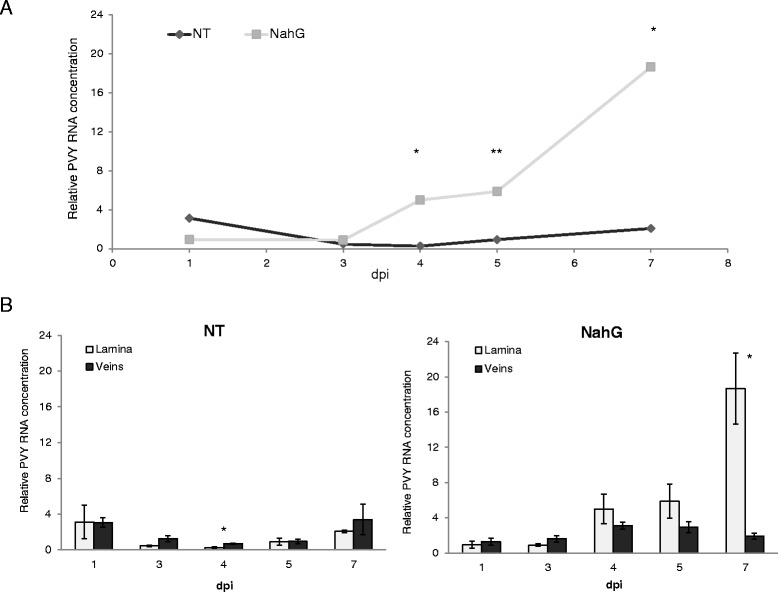
Multiplication of PVY^NTN^ RNA in inoculated potato leaves. **a** Measured viral RNA concentration in nontransgenic plants (NT, dark grey) and NahG-Désirée potato plants (NahG, light grey). **b** Viral RNA concentration measured separately in lamina (light grey) and veins (dark grey) of NT (bottom left) and NahG plants (bottom right) of cv. Désirée. Relative PVY RNA concentration normalized to reference genes (cox and 18S rRNA) expression (±SE, *n =* 3) in the inoculated leaves is given for differences between genotypes (NT *versus* NahG): * - *p <* 0.05, ** - *p <* 0.1, dpi – days post inoculation. Detailed results of ANOVA are given in Additional file 2

Potato-PVY^NTN^compatible interaction was investigated in the tolerant potato cv. Désirée and the role of salicylic acid was evaluated using transgenic NahG-Désirée. The only symptoms observed in virus-inoculated plants of cv. Désirée compared to mock-inoculated plants was slightly more yellowing of the inoculated leaves starting at 7 days post inoculation (dpi) (Additional file 1A). Although Désirée plants did not appear to be affected, the PVY^NTN^ multiplied in the inoculated leaves from 5 dpi on (Fig. 1a, Additional file 2) and the spread of viral RNA to upper leaves was previously detected at 7dpi [13].

In contrast to the nontransgenic plants of cv. Désirée, the SA-deficient transgenic NahG-Désirée showed a greater susceptibility to PVY^NTN^ inoculation. We observed round necrotic and chlorotic lesions on inoculated leaves beginning from 4 dpi, as well as vein necrotic lesions. Both symptoms became more pronounced in later dpi (Additional file 1A). The appearance of the symptoms in NahG-Désirée corresponded to the first detection of viral multiplication at 4dpi (Fig. 1a, Additional file 2). These results confirm important role of salicylic acid in restricting viral spread since its lack causes stronger symptoms development. They also correspond to our previous observations [13].

Moreover viral multiplication in the leaf lamina and veins was measured separately (Fig. 1b). Statistically significant differences (* - *p <* 0.05, ** - *p <* 0.1) between viral RNA accumulation in lamina and veins were evaluated. In NT Désirée plants, viral RNA was relatively evenly distributed between the veins and the lamina, except at 4 dpi when the concentration of viral RNA was slightly yet significantly higher in veins than in the lamina (Fig. 1b left).

In SA-deficient plants, the amplification of viral RNA in the lamina was detected in parallel with the appearance of symptoms (4 dpi), while the viral RNA concentration in the veins remained mostly unaffected throughout the analyzed time course (Fig. 1b right). It is known that plant viruses exploit phloem as the route of long-distance movement and systemic infection in host plants [15] even though the exact mechanism for PVY transport remains unclear [16]. Since the viral concentration in veins of NahG-Désirée did not increase during the disease development, these results could suggest that the vein tissue is mainly used for viral spread rather than its amplification. In the lamina however a high accumulation was detected, suggesting that this could be the place where viral multiplication predominately takes place.

Callose accumulation was also evaluated (Additional file 1B) as it was previously implicated to have an important role in virus spread [17–19]. Callose is believed to be one of the efficient regulators of plasmodesmal permeability, and its deposits are believed to act as a physical barrier to the spread of viruses [20]. We evaluated the dynamics of callose accumulation in both potato genotypes. No deposits were observed in Désirée plants neither at the infected nor at the control plants. Callose accumulation was evaluated also in SA-deficient plants. Only infected NahG-Désirée leaves started to accumulate callose in lesions and around necrotic veins, while the surrounding tissue remained unchanged (Additional file 1B). The timing of callose accumulation corresponded to the time when first lesions appeared (at 4 dpi) suggesting its linkage to symptoms developed. The absence of callose accumulation as efficient barrier in NT plants corresponds with more or less equal distribution of viral RNA between lamina and veins (Fig. 1b). Similarly in NahG genotype where callose was accumulating around the lesions, viral RNA was found to be much more expressed in lamina than in the veins.

A study of hypersensitive resistance response of potato cv. Rywal (where viral spread is completely prevented) showed that callose deposition border and separate the lesion cells from the surrounding tissue, from 3 dpi [21]. In our study callose depositions demarcate the lesion cells from the surrounding tissue in NahG-Désirée, however, not determining in the resistance, since the viral multiplication was not restricted.

### Transcriptional dynamics of light reactions-related genes

**Fig. 2:**
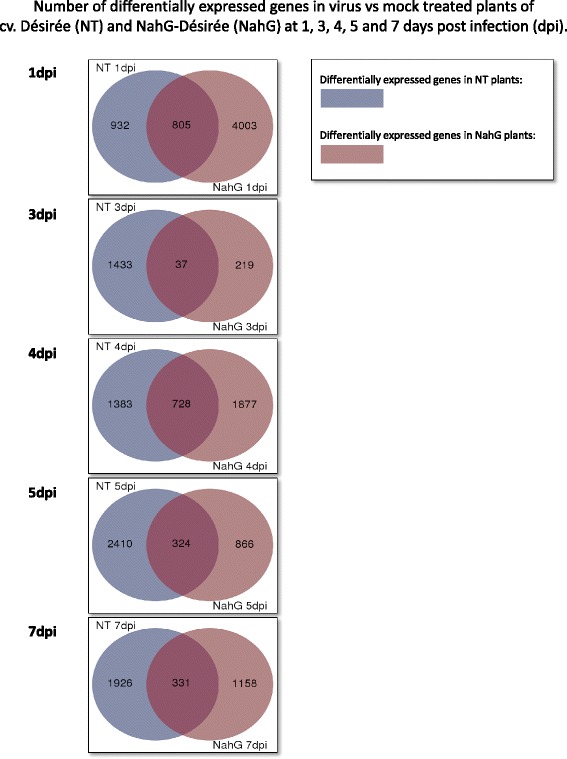
Venn diagram of differentially expressed (DE) genes. Differentially expressed (DE) genes in virus *versus* mock treated plants of cv. Désirée (NT) and NahG-Désirée (NahG) at 1, 3, 4, 5 and 7 days post infection (dpi) are shown. The values represent statistically significantly (FDR p-value <0.05) differentially expressed genes. POCI IDs (Potato Oligo Chip Initiative) [64] have been translated stNIB IDs [78] to represent unique genes. All together 37,865 transcripts have been detected

Whole genome expression analysis was performed to obtain a global overview of the transcriptional dynamics in potato infected with PVY^NTN^. Transcriptome analysis was performed on plants inoculated with PVY at 0, 1, 3, 4, 5 and 7 dpi and compared to the control plants for Désirée and NahG-Désirée plants. Viral infection induced extensive changes in gene expression (Fig. 2, Additional file 3). Venn diagrams demonstrate that most of these changes were not preserved between Désirée and NahG-Désirée plants showing the importance of SA-signaling in plant defense.

Gene-set enrichment analysis was preformed to obtain a summary of the changes at the level of processes (Table 1). Within this study, we focused on analysis of the expression dynamics for genes involved in primary metabolism; photosynthesis and sugar metabolism (Additional file 3). The microarray results were validated by measurement of the expression levels of eight biologically relevant genes using RT-qPCR. A high correlation (0.74) between the data obtained by these two methods was achieved (Additional file 4). A separate report that will report on the dynamics of defense signaling-related responses is in preparation.

Dynamic regulation of genes has been observed at all levels of photosynthesis (Table 1, Additional file 3). Genes coding for thylakoid proteins have previously been shown to be highly responsive to biotic stress [11, 22]. A meta-analysis of the effects of different biotic agents, including arthropods, fungi, and bacteria, and viral pathogens, revealed a near global down-regulation of genes that code for proteins in the reaction centers of photosystems I (PSI) and II (PSII), for ATP synthase, and for several elements of the light-harvesting complex that is associated with PSII (LHCII) [11]. Our study shows that these genes can be differently regulated at specific stage of infection. In cv. Désirée, viral infection strongly affected genes that code for PSII (Table 1, Additional file 3). Many chlorophyll a and b binding protein mRNAs were induced in the first days of viral infection (Table 1, Additional file 3). The products of these genes attach the chlorophyll and antenna pigments to the thylakoid membrane, to form the P680 reaction center of PSII [22]. Their strong induction was detected especially at 1 dpi and 4 dpi (Table 1), and the same trend was observed at 3 dpi on the level of single-gene expression (Additional file 3). After the initial activation, down-regulation of PSII-related genes was detected at 5 dpi (Table 1). Similar effects were observed for the genes that code for PSI protein complexes, with initial activation, followed by down-regulation. This strong shift in gene expression coincided with the detection of viral multiplication at 5 dpi (Fig. 1). Transient activation of PSII and PSI implies higher energy demands needed for plant defense. The following decrease in expression of both photosystems is a sign of allocating the resources and leading to metabolic transition from source to sink in infected leaves.

On the other hand, genes that code for electron carriers that are involved in electron flow from PSI were repressed throughout the study period (1–7 dpi; Additional file 3). The levels of ferredoxin and ferredoxin reductase mRNAs were significantly decreased in PVY-treated plants compared to controls (Table 1, Additional file 3). Several other studies, however, have reported both down-regulation and up-regulation of specific ferredoxin and ferredoxin reductase genes in response to different pathogens, and it was suggested that the up-regulation of these genes reflects their involvement in redox processes of plant defense responses or direct interactions between pathogen elicitors and these proteins (reviewed in [11]). Recently it was shown that the coat protein of tomato mosaic virus (a member of the genus Tobamovirus) interacts with ferredoxin [23]. Interactions between potyviral HC-Pro protein and ferredoxins have also been confirmed in maize and sugarcane mosaic virus interaction [24]. The authors suggested that this interaction might lead to impaired import of ferredoxins to chloroplasts, and thus contribute to the development of chlorotic symptoms in host plants and/or to perturbations in chloroplast structure and function [23].

We showed that virus infection strongly affects photosynthesis-related gene expression. The initial induction of both photosystems was diminished in later days post infection. This transient activation was present only at the level of PSI and PSII. At the level of electron flow from the PSI only repression was detected. Taken all the data together we see dynamic, pulse activation of many genes involved in photosynthesis. Such regulation of transcriptional activity has been previously noted in the process of plant defense [25]. A model of transcriptional cascades as part of defense network and sequential pulse activation of specific genes via fine-tuned ROS signaling and phytohormonal bursts was proposed [25]. Similar mechanism could be the basis of our observed virus induced-activation of light reaction-related genes but more detailed spatio-temporal analysis including perhaps also ROS species as well as hormones would be needed to confirm that.

### Subunit-specific regulation of RuBisCO transcripts

Calvin cycle is the most common pathway for carbon fixation in plants [26]. Our data show that transcripts involved in this pathway are also dynamically regulated, and they follow the general trend of photosynthetic electron-transport reactions with a mild delay. At the level of process analysis, we observed induction of the Calvin cycle at 3 dpi, and repression at later times (5 dpi and 7 dpi) (Table 1). For some specific reactions within the Calvin cycle, constant repression has been observed at the level of gene expression (genes that code for the glyceraldehyde-3-phosphate dehydrogenases, fructose-bisphosphate aldolases, and phosphoribulokinases), while other genes were induced throughout the study (genes that code for phosphoglycerate kinase and triose-phosphate isomerise) (Additional file 3, Additional file 5). These data shows that there is a specific regulation of individual reactions of carbon fixation.

**Fig. 3:**
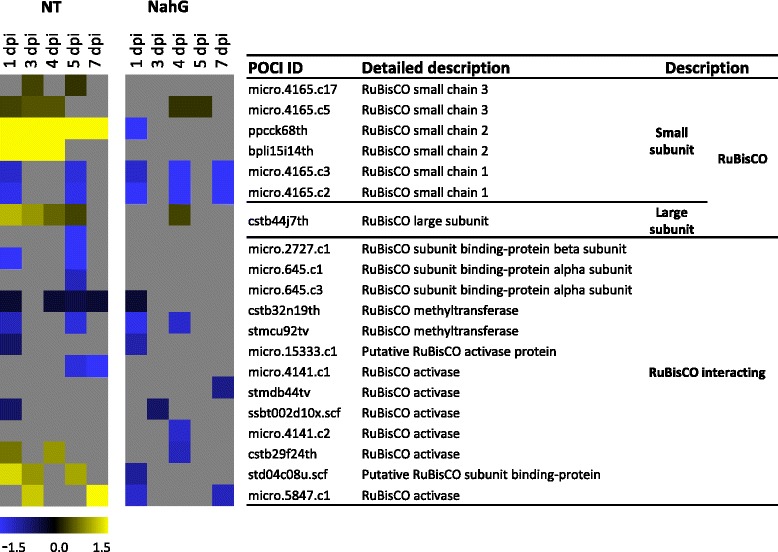
RuBisCO transcripts are specifically regulated in virus-induced responses. Gene expression values of RuBisCO small and large subunits and its regulators were log_2_ transformed and a fold change difference (log_2_FC) was calculated for PVY *versus* mock. Only statistically significant differences between PVY and mock inoculated leaves are presented (FDR corrected p-value < 0.05). The results for two genotypes are shown: NT - nontransgenic plants of cv. Désirée, NahG – transgenic plants of cv. Désirée overexpressing NahG. Yellow-up-regulated genes; blue-down regulated genes; grey-no statistically significant difference in gene expression

The central enzyme for the assimilation of atmospheric carbon dioxide into organic compounds is the highly abundant ribulose 1,5-bisphosphate carboxylase/oxygenase (RuBisCO) [27, 28]. In higher plants, the complete and functional RuBisCO is composed of 16 subunits: eight small subunits, and eight large subunits [29–32]. The large subunits are encoded in the chloroplast genome [33], while the small subunits are encoded in the nucleus and are transported into the chloroplast [34]. For stoichiometric assembly, equimolar concentrations of both of these subunits are required. In the present study, we identified differential expression of three small subunits-encoding genes; RuBisCO small chain 1, 2 and 3 (Fig. 3). Each of them has an individual expression pattern. While small subunit 1 was strongly significantly repressed at 1 and 5 dpi, small chains 2 and 3 were either strongly (RuBisCO small chain 2) or fairly (RuBisCO small chain 3) induced thorough the time course (Fig. 3). Interestingly, RuBisCO large subunit followed the expression pattern of small chains 2 and 3 and was induced from 1 dpi to 5 dpi. RuBisCO regulators which influence RuBisCO’s activity (the RuBisCO activases and methylases) were also found to be dually regulated similarly to the RuBisCO small subunits, with some of the transcripts being induced and other repressed (Fig. 3).

An additional role of the RuBisCO small subunit was reported for *Tomato Mosaic Virus* (ToMV, *Tobamovirus*) infected *Nicotiana benthamiana* [35]. This study showed that movement protein of ToMV interacts with the RuBisCO small subunit. The silencing of the RuBisCO small subunit gene induced necrosis in inoculated leaves, and enabled the generation of more infection sites. This interaction is also important in tobamovirus movement [35]. Interactions between potyvirus proteins (PIPO) and both the RuBisCO small subunit and the RuBisCO large subunit were also detected, and these interactions are believed to contribute to symptom development in host plants [36]. Induction of the small and large RuBisCO subunits might compensate for the loss of activity caused by the virus, and thus contribute to the asymptomatic status of the nontransgenic Désirée plants. The dynamics of host photosynthesis gene expression have been studied previously over shorter time-course experiment for the hypersensitive resistance response of potato to PVY [21]. Interestingly, expression of the RuBisCO small subunits was induced in early phases of responses (at 1 dpi and 3 dpi) [21].

### Transcriptional changes in sugar metabolism-related genes

The trend of the dynamics of sugar accumulation is globally inclined toward the induction of genes involved in the synthesis of starch. At 1 dpi, the viral infection did not cause changes in sugar metabolism (Table 1), whereas at 3 dpi, the transporters involved in the synthesis of starch, such as ADP/ATP carrier proteins (i.e., adenine nucleotide translocators) and glucose-6-phosphate translocators were significantly activated and continued to be induced up to 7 dpi (Additional file 3). At the same stage, the transcriptional responses indicated the induction of virus-stimulated degradation of sucrose (Table 1), which was mainly due to induction of some fructokinases and sucrose synthase 4 (Additional file 3). A transient induction of galactose metabolism at 3 and 4 dpi, was the only virus-induced change detected at the level of minor carbohydrate metabolism (Table 1).

**Table 1:** Dynamics of primary metabolism-related transcriptional responses

Process description (MapMan BIN)	No. of genes in BIN	NT					NahG				
		1 dpi	3 dpi	4 dpi	5 dpi	7 dpi	1 dpi	3 dpi	4 dpi	5 dpi	7 dpi
Photosynthesis	354		+	+	-		-		-		-
Light reaction	208	+		+	-		-		-		-
Light reaction.photosystem II	108	+		+	-		-		-		-
Light reaction.photosystem II.LHCII	25	+		+	-		-		-		-
Light reaction.photosystem II.PSII polypeptide subunits	83	+		+	-		-		-		-
Light reaction.photosystem I	43	+		+	-		-	+	-		-
Light reaction.photosystem I.Psi polypeptide Subunits	26			+	-		-	+	-		-
Light reaction.other electron carrier	21	-			-	-	-		-		-
Photorespiration	44		+		-		-		-	-	-
Calvin cycle	102		+		-	-	-		-	-	-
Major cho metabolism	227		+	+		+					
Major cho metabolism.synthesis	97			+	-	+					
Major cho metabolism.synthesis.starch	82			+	-	+					
Major cho metabolism.synthesis.starch.starch synthase	27					+					-
Major cho metabolism.synthesis.starch.transporter	23		+	+		+					+
Major cho metabolism.degradation	130		+				+		+		
Major cho metabolism.degradation.sucrose	58		+				+				+
Major cho metabolism.degradation.sucrose.invertases	20						+	-			+
Major cho metabolism.degradation.starch	72	+		+		+		+	+		
Major cho metabolism.degradation.starch.starch cleavage	35						+		+		+
Minor cho metabolism	185										
Minor cho metabolism.trehalose	31						+				-
Minor cho metabolism.others	75							+			
Minor cho metabolism.galactose	20		+	+							
Glycolysis	106										

Virus-induced changes on the level of process analysis are shown for primary metabolism- related processes (photosynthesis, major and minor sugar metabolism and glycolysis). Results of Gene set enrichment analysis using MapMan ontology at 1, 3, 4, 5 and 7 dpi are presented in potato cv. Désirée (NT) and NahG-Désirée (NahG). Only statistically significant (FDR p-value <0.10) enriched sets of genes are marked as induced (+) or repressed (-) process. dpi – days post inoculation, Process description – according to MapMan gene annotation. No of genes in BIN – number of all genes assigned to the BIN. Blanks denote that a current process was not statistically significantly enriched at a corresponding dpi. Visualization of these results in primary metabolism pathways is presented in Additional file 5

Over the days examined, there were no virus-induced changes in the transcriptional regulation of genes involved in glycolysis (Table 1), which suggested the stability of this part of the metabolic processes.

For many years, it has been suggested that the role of primary metabolism during plant-pathogen interactions is to support cellular energy requirements for plant defense responses [37, 38]. The trade-off between plant growth and immunity was shown to be finely regulated to ensure proper allocation of resources [39]. More recent studies have linked carbohydrate metabolism directly with plant defense responses [reviewed in 36]. Experiments demonstrated induction of PR genes by sugars such as glucose, fructose and sucrose even in the absence of pathogens. Further evidence for the role of carbohydrates in defense responses has been provided by experiments showing the induction of genes (such as cell wall invertase, hexokinase and glyceraldehyde dehydrogenease, pyruvate dehydrogenase, ribose-5-phosphate isomerase etc.) involved in carbohydrate metabolism upon challenge by pathogens [40]. Our results have made it clear that there is an abundance of virus-induced expression of genes involved in primary metabolism which are fine tuned in a timely manner. That regulation of sugar-mediated defense responses is complex and it operates at multiple levels.

### Salicylic acid depletion diminishes bimodal expression patterns of light reaction-related genes

Our analysis have supported the role of SA as an important player in the plant defense response, and confirmed that perturbation of SA signaling severely affects the status of the plant, by allowing more efficient viral accumulation and pronounced symptom development ([13]; Fig. 1, Additional file 1). Therefore, to determine the impact of SA in plant responses, a whole genome analysis was also performed on the SA-deficient transgenic plants NahG-Désirée. Many virus-induced gene expression changes differed between NT Désirée and NahG-Désirée plants (Fig. 2). Through the time investigated only a subset of transcriptional changes was the same in both genotypes (Fig. 2). Comparing the primary metabolism—related gene expression between NT and SA-deficient Désirée plants revealed major differences, implying an important role of SA signaling in virus-induced photosynthesis and sugar metabolism (Table 1).

Plants lacking SA signaling respond to viral infection with general inhibition of photosynthesis-related genes. Reduction of photosynthesis genes involved in light reactions, photorespiration and Calvin cycle was detected at 1 dpi, 4 dpi and 7 dpi, while interesting at 3 dpi and 5 dpi most of the genes remained unresponsive (Table 1). Viral infection of NahG-Désirée caused suppression of the light-dependent reaction-related genes at 1, 4 dpi and 7 dpi. The induction of light reaction genes in NT Désirée plants early after infection was thus shifted to inhibition when plants were lacking SA (Table 1), suggesting that SA-deficiency affects light reaction mostly at 1 and 3 dpi. The second repression detected at 5 dpi in NT Désirée plants was observed in NahG plants only at 7 dpi.

Looking more specifically into different light reaction components, SA-deficiency strongly affected the virus-induced expression profiles of PSI—and PSII-related genes. At 1 dpi and 4 dpi, the induction of genes coding for both photosystems detected in nontransgenic plants, was reversed to repression in NahG plants (Table 1, Additional file 5). These genes were again repressed only in pulse manner and remained unresponsive at 3 and 5 dpi (Table 1). We observed more similar expression profiles between both these genotypes for the electron carrier-coding genes (i.e., down-regulation in both genotypes), which indicated that the electron flow from PSI was not substantially affected by the SA depletion (Table 1, Additional file 3). The role of SA on photosynthesis has been previously noticed. In hypersensitive resistant potato cultivar Rywal, PVY multiplication in the NahG plants was accompanied by down regulation of photosynthesis genes [21].

**Fig. 4:**
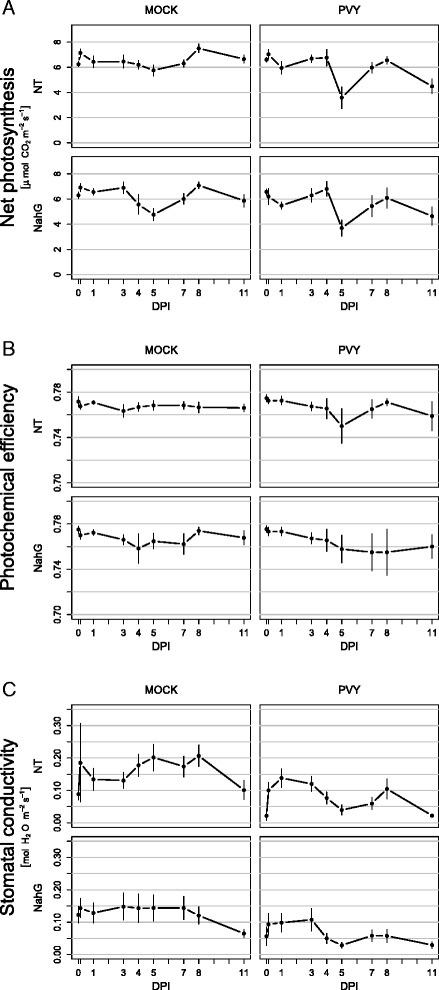
Dynamics of photosynthesis in tolerant potato-PVY^NTN^ interaction. Net photosynthesis (**a**), photochemical efficiency (**b**) and stomatal conductivity (**c**) of potato leaves at different stages of viral infection were measured in nontransgenic plants of cv. Désirée (NT) and NahG-Désirée (NahG) following mock (left panel) inoculation and inoculation with PVY^NTN^ (right panel). Measurements were performed on inoculated leaves. Error bars represent 95 % confidence intervals. Statistical evaluation of data is given in Additional file 2 and the remaining photosynthetic parameters in Additional file 6

General inhibition of photosynthesis genes implies a metabolism of reduction of energy production suggesting that NahG infected leaves became a sink tissue since the first day after the infection. However the repression happens in a pulse manner with unresponsive days. Additionally, transcriptional dynamics was not in accordance with measured photosynthetic rate (see next section). First transcriptional down regulation of photosynthetic genes at 1 dpi preceded the reduction of measured net photosynthesis detected at 5 dpi (Fig. 4).

SA-signaling also affected virus-induced changes in sugar metabolism. Differences between the nontransgenic and NahG-Désirée plants were detected for genes involved in major and minor carbohydrate metabolism. The induction of starch synthesis-related genes was diminished in the SA-deficient plants (Table 1). In these leaves most of the virus-induced changes of major sugar metabolism were detected in the process of degradation of sucrose and starch. Sucrose is the main form of assimilated carbon which is produced during photosynthesis and then transported from source to sink tissues via the phloem. It affects growth and development, differential gene expression and stress-related responses and it was shown to act as an endogenous signal to induce defense responses against pathogens [41–43]. Invertases are affecting the sucrose: hexose ratios linked to sugar signaling [41, 44] and are also markers of sink/source status of the tissue [45]. Expression pattern of invertases was affected only in NahG plant (Table 1), however no major effect on cell wall invertases, that are commonly linked to plant defense against pathogens [43] and source/sink status has been detected (Additional file 3).

NahG plants have induced transcription of genes involved in starch degradation (Table 1, Additional file 3) possibly due to altered need for energy resources in the leaves or again as a result of sugar-mediated signaling defense mechanisms. This induction has been observed only at minor extend in nontransgenic plants (Table 1, Additional file 3).

Comparing both genotypes we observed the differences in expression of genes involved in trehalose metabolism (Table 1). Trehalose is another well-known non-reducing sugar that has been shown to link primary metabolism and stress, e.g. it induces resistance against powdery mildew in wheat by activation of phenylalanine ammonia-lyase, an enzyme that is involved in biosynthesis of SA [46].

Besides the known effect of photosynthesis efficiency on sugar metabolism it is also known that carbohydrate accumulation in leaves may result in a reduction in photosynthetic rates by direct or indirect feedback inhibition mechanisms [47]. However due to diverse dynamics on different levels of energy producing metabolism it is difficult to disentangle the effects between sugar metabolism and photosynthesis based on information obtained in this study. We showed that SA influences virus-induced gene expression on many levels of primary-metabolism, that the effect is diverse, complex and strongly temporally dependent with pulse activation of genes.

### Chlorophyll fluorescence confirms that PVY infection inhibits the light photosynthetic reactions

Transcriptome analysis revealed an intensive response of the photosynthetic genes to PVY infection. We additionally examined whether these changes are reflected in the efficiency of photosynthesis.

In the nontransgenic Désirée plants, the initial induction of light-reaction genes at 1 dpi, 3 dpi and 4 dpi was sharply reversed to suppression at the time corresponding to the onset of detectable viral multiplication (5 dpi) (Table 1, Additional file 3). Similarly, a decrease in the photochemical efficiency was observed at 5 dpi (Fig. 4b). This fluorescence parameter that is largely attributed to the efficiency of PSII [48], was relatively unresponsive and stable initially, but transiently decreased at 5 dpi in the PVY-infected plants. Reduced photochemical efficiency has also been reported in other studies that used different fluorescence techniques to study the effects of PVY in tobacco [49–51]. Short time-series measurements revealed reduced efficiency of the electron donation to the PSII reaction centers, and decreased rates of photosynthetic quantum conversion at the PSII reaction centers [51].

In general, these effects of virus on PSII can have different mechanistic explanations. In the case of *Tobamovirus Tobacco Mosaic Virus* (TMV), a direct interaction of the viral coat protein with PSII proteins was demonstrated, as an inhibitory association [52]. No direct interactions between potyviral proteins and the PSII proteins has however been confirmed to date [53]. It has also been suggested that the effects on PSII derive from the lowered efficiency of the protein-repair mechanism due to extensive synthesis of viral proteins, or from impaired transport of nuclear-encoded chloroplast proteins into chloroplasts [51]. Our transcriptomic data, however, indicate intensive responses of genes coding for proteins involved in light harvesting and oxygen evolution at PSII (Table 1, Additional file 3), which fits well with the data from our fluorescence measurements. One of the possible mechanisms that lead to lower photochemical efficiency might thus also simply be the lower production of PSII components. A correlation of responses at the transcriptional level and at the level of fluorescence is not trivial, as reduced expression of photosynthetic light-processing genes does not necessarily translate to loss of function. The temporal relationship between the expression of ‘light-reaction’ genes and the function of the process is not immediate, because of the long functional lifetime of the thylakoid proteins. Moreover, their production and assembly are not often proximally regulated by transcription [11]. This could explain the relatively low responsiveness of the thylakoid function to PVY (4 % reduction in photochemical efficiency; Fig. 4b).

Similar to the nontransgenic plants, in the NahG-Désirée plants, there was a significant decrease in photochemical efficiency that started at the time of viral multiplication (4 dpi), although contrary to the nontransgenic plants, this persisted until the end of the measurements. These differences might reflect distinct transcriptional regulation of the genes involved in light reactions, which were initially up-regulated in the nontransgenic plants, but mostly down-regulated in the NahG-Désirée plants (0 dpi to 7 dpi). In addition, the differences might be attributed to the development of the symptoms (necrotic lesions and vein necrosis; Additional file 1) that appeared in the NahG-Désirée plants but not in the nontransgenic plants. The next decrease in the actual photochemical efficiency ratio (F_v_’/F_m_’) at 11 dpi was detected in all of the plants, and coincided with the decrease of leaf chlorophyll (measurement of SPAD; Additional file 6). As the decrease in these parameters was observed in both infected and mock inoculated plants we consider it as senescence phenomena which occurred due to aging of the plants and not as a consequence of viral infection.

### PVY infection transiently reduces the net photosynthetic rate at the onset of detectable viral multiplication

In comparison to the fluorescence measurements that allow for a direct insight into the quality of the thylakoid processes, the complexity of the photosynthetic physiology is integrated in the parameters obtained using gas-exchange measurements. Analysis of the transcriptome showed that PVY can have both stimulatory and/or inhibitory effects on different parts of primary metabolism (Table 1, Additional file 5). As well as an effect on photosynthetic light and carbon reactions, changes in the processes such as photorespiration (strongly affected at the level of transcription and the proteome; see next section), respiration, sucrose and starch metabolism (influencing sink–source relations) and other processes are translated into the end result—a given rate of net photosynthesis (P_n_) (P_n_ = gross photosynthesis—respiration—photorespiration). Despite this complex integration, the temporal patterns of the gas-exchange data shared some characteristics of the fluorescence parameters. A decrease in net photosynthesis at 5 dpi in both of the genotypes studied was observed (Fig. 4a and Additional file 2).

The reduction in photosynthesis is a general response to pathogen attack, although sometimes induction was also detected (e.g. [54]). For PVY infections, a decrease in Pn was reported in sensitive *Nicotiana tabacum* cultivars [49–51]. A recent study that analyzed the photosynthetic responses at three different stages after infection showed that Pn and other related parameters, such as stomatal conductivity and transpiration, were not affected at 3 dpi, but decreased later at 7 dpi to 14 dpi [51]. Similarly, our measurements showed reductions in Pn in both symptomatic and asymptomatic plants. Additionally, more detailed time-series analysis revealed relatively dynamic, nonlinear responses of the gas-exchange parameters during the development of infection (Fig. 4b).

The reduced net photosynthesis of the PVY-infected nontransgenic and NahG-Desiree plants coincided with decreased stomatal conductivity (Fig. 4c, Additional file 7), which suggested that the photosynthetic process was not influenced only by the PVY effects on the photosynthetic reactions. It might be limited by inadequate diffusion of the photosynthetic substrate CO_2_ to the leaf, which is a far more indirect effect related to regulation of the stomata. Also, other studies have reported that the PVY-induced reduction of P_n_ is a consequence of several factors, including a decrease in RuBisCO activity and the content of chlorophylls and xanthophyll cycle pigments contents, an impairment of photosynthetic light reactions, and stomatal closure [50]. It is likely that the viral infection induces changes in the plant metabolism that interfere with signal transduction mechanisms participating in stomatal regulation. Indeed, in connection with stomatal regulation, the importance of some other hormones have recently been recognized, including jasmonic acid, in addition to abscisic acid [55]. Our previous studies showed that not only SA but also the signaling of other hormones, like jasmonates, ethylene, auxines and abscisic acid, is regulated in potato-PVY interaction [21, 56], which could also result in the regulation of stomatal conductance.

### Changes in the leaf proteome following PVY infection

**Fig. 5:**
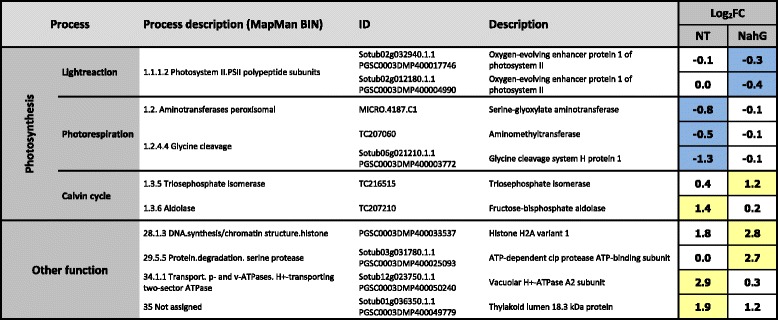
Virus-affected protein abundance. The table shows proteins whose expressions have been up-regulated or down-regulated due to PVY infection. Proteins are grouped according to their function, as determined by MapMan ontology. Samples collected at 4 dpi were analyzed. The average ratios (log_2_FC) of the protein abundance in PVY *versus* mock inoculated plants are shown. Only proteins that showed statistically significantly differentially expression (***p <***0.05) in at least one genotype are included. Significance is marked with shading; Blue - significantly decreased proteins, yellow - significantly induced proteins. NT – cv. Desiree; NahG – NahG-Désirée

To bridge the gap between the transcriptional regulation and the plant status analyzed with the gas-exchange and fluorescence measurements, we analyzed a snap-shot of the response at the level of protein accumulation. Samples of both genotypes (nontransgenic and NahG-Désirée) were analyzed at 4 dpi. This is the time when the PVY multiplication was first detectable in the NahG-Désirée plants, 1 day before the shift in the light reaction-involved gene expression, and 1 day before the measured decrease in net photosynthesis rates for both genotypes (Fig. 4a). We identified and quantified 339 proteins in the potato leaves that were mainly involved in photosynthesis: i.e., in light reactions, photorespiration and the Calvin cycle (Additional file 8). Eleven of these proteins were statistically significantly differentially abundant after infection with PVY in at least one of the genotypes studied (*p <* 0.05) and are visualized in Fig. 5. The results of full factorial analysis of variance show that abundance of 10 proteins is also SA-dependant (Additional file 8).

In the nontransgenic plants, the viral infection resulted in significantly lower abundances of the proteins involved in photorespiration. Serine glyoxylate aminotransferase, aminomethyltransferase, and glycine cleavage system H protein were down-regulated at 4 dpi (Fig. 5), which corresponded to the global trend of the aminotransferase mRNA expression patterns acting in photorespiration (Additional file 3). Virus infection increased fructose-bisphosphate aldolase concentration, suggesting a virus-induced activity of Calvin cycle. Activation of Calvin cycle-related transcripts has been detected at 3 days post viral infection and with a time shift of 1 day this effect is reflected also on protein level. Among proteins classified to be involved in other functions as photosynthesis, we identified an up regulation of vacuolar H + -ATPase, and thylakoide lumen protein. The role of pathogen-dependent activation of H + -ATPase has previously been investigated in *Lycopersicon esculentum* plants infected with *Cladosporium fulvum* [57]*.*

Protein analysis has once again proven the important role of hormonal regulation of plant defense. Salicylic acid deficiency has caused major differences in protein abundance profiling. None of the significant virus-induced changes observed in nontransgenic plants were found in SA deficient plants. In the virus-infected NahG-plants, there were lower levels of PSII-associated oxygen-evolving enhancer protein 1 detected. This repression corresponded to the measurements at the level of the transcripts, where the same trend of down-regulation in the expression of the PSII-related genes was observed at 4 dpi (Additional file 3). As in the nontransgenic Désirée plants, the Calvin cycle was also affected in the NahG-Désirée but this time at the point of conversion of glyceralaldehyde-3-phosphate to dihydroxyacetone-3-phosphate with higher abundance of the enzyme triosephosphate isomerase (Fig. 5). In addition to photosynthesis-related proteins, differential abundance of proteins involved in other functions has also been detected (Fig. 5). Virus-dependent induction of histone H2A and CLP protease was shown to work in a SA signaling-dependent manner.

Not all of the changes observed at the level of the transcriptome were also confirmed at the proteome level (Additional file 9). One of the reasons is that the regulation of gene expression is decoupled from protein translation. The proteome is in general more stable, and only some of the transcriptional perturbations are reflected at the level of the proteins, due to posttranscriptional regulation mechanisms or differences in protein turn-over rates [58, 59]. Additionally, discrepancies have also been commonly observed in comparisons of proteome and transcriptome data over time, and these might be the consequence of a time delay between transcript and protein synthesis [21, 60, 61]. The discrepancies might also be the consequence of technical limitations in the proteomics. Microarrays enable analysis of the expression of all 40,000 genes in parallel, while shotgun mass spectrometry does not allow for the identification of all proteins, but usually only covers a range of 300 to 2,000 proteins depending on the species [62].

## Conclusions

Photosynthesis-related gene expression profiles revealed that the potato response to PVY infection is far from being a static induction of key components. Instead, strong dynamics in the responses are observed. Not only do the transcripts affected respond in a rapid manner, with the fast transition from induction to repression, but there also appears to be fine-tuned regulation of subsets of the genes involved in the process. The induction of a process, or even a metabolic pathway, can shift to strong repression in 24 h, as for the photosynthesis light reactions from 4 dpi to 5 dpi in cv. Désirée. This was reflected in the drop in photosynthetic efficiency, as well as the integrated net photosynthetic rate at 5 dpi in the PVY-infected leaves. The timing of this major metabolic shift corresponds to the onset of detectable virus multiplication. The differences in the gas-exchange parameters were transient, which indicated a relatively large compensatory capacity of the photosynthetic metabolism, and potentially of the other levels of regulation. The PVY-induced transcriptional activation of photosynthesis-related genes in the first days following infection was, however, buffered, and an increase in the photosynthetic activity was not observed.

Down-regulation of the photosynthetic machinery was similarly observed in the SA-deficient potato plants however the induction of several photosynthesis-related genes early in the infection was diminished in the transgenic plants. As some of these components have already been shown to interact with viral proteins, the overproduction of host proteins might contribute to the absence of symptoms in cv. Désirée. Major alterations of the defense response at the level of transcriptome, proteome as well as at the level of photosynthesis performance demonstrated the important role of the SA phytohormone in potato defense.

## Methods

### Plant materials

Potato (*Solanum tuberosum* L.) cv. Désirée and transgenic potato plants expressing SA hydroxylase (NahG-Désirée) were propagated in tissue culture. Two weeks after node segmentation, they were transferred to soil in a growth chamber, and kept at 21 ± 2 °C in the light and 18 ± 1 °C in the dark, at a relative humidity of 75 % ±2 %, with 70-90 μmol/m^2^/s^2^ radiation (L36W/77 lamp, Osram, Germany) and a 16-h photoperiod. After 4 weeks of growth in soil, the potato plants were inoculated with PVY^NTN^ (isolate NIB-NTN, AJ585342) as described in [63]. For the mock-inoculated plants, the same procedures were performed with sap from healthy potato plants.

On the day of the inoculation, three leaves from three nontreated plants for each genotype (Désirée, NahG-Désirée) were collected, which were designated as controls (0 dpi). PVY^NTN^-and mock-inoculated leaf samples were collected on 1, 3, 4, 5 and 7 dpi. Three plants for each treatment were used. The midvein was cut out, and both the lamina and the vein were sampled separately and then directly frozen in liquid nitrogen and stored at –80 °C.

### Microarray analysis

Total RNA from the inoculated leaves was extracted, DNase treated, purified, and quality controlled as described previously [56]. A one-color based hybridization protocol was performed on the custom 60-mer oligo microarrays (4 × 44 K; AMADID 015425) designed by the Potato Oligo Chip Initiative [64]. For each sample at least 1 μg total RNA was used and sent for analysis at IMGM Laboratories GmbH, Germany. The raw data were analyzed in the R Project for Statistical Computing program (R Development Core Team, 2011; version 2.13.2), using the packages Agi4x44PreProcess [65] and Limma [66].

The microarray features were filtered according to the Agilent quality control flags: if the feature was determined to be well above background, if the noise did not exceed a threshold, and if it was not saturated (IsNOTWellAboveBG, IsSaturated and IsFeatNonUnifOL [65]) in at least 10 % of the total microarray count, then that particular microarray feature was retained for further analysis. The raw data of the remaining 37,865 (from a total of 42,034) features was robust spline normalized (‘rsn’; [67]). The empirical Bayes method [66] was used to detect differentially expressed genes between PVY^NTN^-and mock-inoculated plants at each time point and for each genotype ([68]; corrected p ≤0.05). Functional analysis of differentially expressed genes was performed using the MapMan software tool [69] using ontology adapted for potato [70]. Gene Set Enrichment Analysis [71] was performed (false discovery rate corrected q ≤0.05; bin size between 20 and 400 genes/bin). The data discussed in this report have been deposited in the NCBI Gene Expression Omnibus, and are accessible through GSE58593 (http://www.ncbi.nlm.nih.gov/geo/query/acc.cgi?acc=GSE58593).

### Quantitative real-time PCR analysis

Relative concentration of the PVY^NTN^ RNA [72] and expression of genes involved in photosynthesis (RuBisCO activase (RA) and chlorophyll a-b binding protein (CAB), sugar metabolism (granule bound starch synthase 1 (GBSS1), β-1,3-glucanases (GluI), (GluII),(GluIII), cell wall invertase (CwINV) and defense responses (pathogenesis-related protein 1b (PR-1b) were analyzed using quantitative real-time PCR. Cytochrome oxidase (Cox [73]) and 18S rRNA (Eukaryotic 18S rRNA TaqMan endogenous control; Applied Biosystems, Carlsbad, CA, USA) were used as reference genes. The primers and probe for CAB were designed as described in [56].

RNA was isolated from all samples using RNeasy Plant Mini kits (Qiagen, Hilden, Germany), according to the manufacturer instructions, but with a modified elution step [56]. DNase-treated (Invitrogen, Carlsbad, CA, USA; 0.1 U/DNase per μg RNA) total RNA (1–2 μg) was reverse transcribed using High Capacity cDNA Reverse Transcription kits (Applied Biosystems, Carlsbad, CA, USA) as described in [56]. The samples were analyzed in the set-up for quantitative real-time PCR analysis as previously described [74], in 5 μl reactions using SYBR Green I chemistry for RA, GBSS1, GluI, GluII, GluIII, CwINV and PR-1b genes, and TaqMan chemistry for viral RNA, CAB and reference genes. The details on primer and probes are given in Additional file 10. The standard curve method was used for relative gene expression quantification, and the transcript accumulation of each gene was normalized to average expression of COX and 18S rRNA [13].

### Gas exchange and fluorescence measurements

Measurements of photosynthesis performance were taken with a Li-6400 (LiCor, Lincoln, USA) measuring system equipped a 6400-40 Leaf Chamber Fluorometer. To perform measurements under the conditions that suit growing conditions, the chamber was mounted on a small tripod and positioned on a shelf of the growth chamber, so there was no need to take plants out of it during the measurements. The measuring conditions were controlled by a Li-6400 console following the pre-measured chamber light, temperature, CO_2_ and humidity values (PPFD of 125 μmol m^−2^ s^−1^, 25 °C, 700 μmol CO_2_ mol^−1^, relative humidity, 65 %-75 %).

Six plants of each treatment (mock-nontransgenic Désirée, mock-NahG-Désirée, PVY^NTN^ nontransgenic Désirée, and PVY^NTN^ NahG- Désirée) were measured daily, from 09:00 to 11:00 AM, from day 0 of infection, onward (0, 0.13, 1, 3, 4, 5, 7, 8 and 11 dpi). The two lower inoculated leaves per plant were examined for each treatment. At each measurement, the leaf was enclosed in the chamber and left to achieve steady-state response, then a saturating light pulse was triggered to induce fluorescence, and, in parallel, the gas-exchange data were stored. The data on net photosynthesis (P_n_), stomatal conductance (Cond), actual photochemical efficiency (Fv’/Fm’), potential photochemical activity (Fv/Fm) chlorophyll content (SPAD) and electron transport rate (ETR) of PVY^NTN^-infected plants were evaluated relative to the corresponding mock treatment (e.g., NP_PVY-NT_/ NP_mock-NT_).

### Proteomics analysis

The PVY^NTN^-inoculated and mock-inoculated leaf lamina samples collected 4 dpi from the nontransgenic Desiree and NahG-Désirée plants were analyzed for the proteomics experiments. Two leaves from three potato plants were sampled and pooled together to produce three biological replicates per treatment. 100 mg of plant material was powdered in liquid nitrogen. The Trizol mini-protocol was used for the extraction of proteins [75]. The protein concentrations in the final extracts was determined using the Bradford assay [76]. Two-hundred μg protein was subjected to digestion and desalting, as described previously in [77]. The protein digest (0.5 μg) was loaded onto a Peptide ES-18 column (15 cm × 0.1 mm; 2.7 μm; Sigma-Aldrich) using a one-dimensional nano-flow LC system (UltiMate 3000, Thermo Scientific) coupled to an Orbitrap LTQ XL mass spectrometer (Thermo Scientific) operated in data-dependent mode. Peptides where eluted using a 60-min gradient from 5 % to 80 % acetonitrile/ 0.1 % formic acid, with a controlled flow rate of 0.3 nL per min [77].

The proteins were identified using the SEQUEST algorithm and Proteome Discoverer (v 1.3, Thermo Scientific). To search the MS data against a FASTA file, we created from downloads the complete set of available potato sequences, as described [78]. *In-silico* peptide lists were generated with the following settings: trypsin as the digestion enzyme and a maximum of three missed cleavages. Mass tolerance was set to 5 ppm for precursor ions and 0.8 Da for fragment ions. Additionally, a decoy database containing reversed sequences was used to estimate the false discovery rate (FDR). Only high confidence (FDR ≤ 0.01 %) peptide identifications with a minimum XCorr of 2.0, and proteins with at least two distinct peptides, were considered.

The datamatrix of the ProteomeDiscoverer, which contained spectral count information, was used for quantitative analysis. Missing values (proteins not identified in the sample) were replaced with 0.5 of the minimum protein expression value [79]. Statistical evaluation of differences in protein quantities was performed using analysis of variance with genotype and viral infection included as the factors. For specific analysis of changes due to viral infection within one genotype Student *t*-test was performed.

### Statistical analysis of time-series data

The results of gene expression analysis (microarrays and quantitative real-time PCR) as well as gas exchange and fluorescence measurements in PVY^NTN^-infected samples were normalized to the average values measured in mock-inoculated samples at each time point.

Several types of statistical analysis were performed on the obtained dataset in the R statistical environment (R Development Core Team 2010). The data were log_2_ transformed and normalized using quantile normalization to obtain comparative expression values for different genes. The following factors were included in the analysis of variance: genotype, viral infection, time after infection.

### Callose staining

To visualize callose deposition in potato leaves, a separate experiment was performed. Potato plants of cv. Désirée and NahG-Désirée were mock- and PVY^NTN^-inoculated and the leaves collected at 0, 1, 3, 4 or 6 dpi. Fragments of leaves were dissected, immediately syringe infiltrated with aniline blue fluorochrome (Biosupplies, Australia), and analyzed using a Leica TCS-SP5 confocal microscope. The acquired images were processed using LAS-AF-lite software.

## Additional files

Additional file 1:
**Symptom development and callose accumulation after PVY** ^**NTN**^ **inoculation.** (A) Phenotypes of the PVY^NTN^-inoculated (right panel) and mock-inoculated plants (left panel). Symptoms on PVY^NTN^-inoculated leaves are shown at 7 and 11 dpi, and compared to control mock inoculated leaves for cv. Désirée (upper) and NahG-Désirée (bottom). (B) Callose deposition stained with aniline blue. Leaves of NT Désirée and transgenic NahG-Desiree were either PVY or mock-inoculated. In NT Désirée plants (NT- PVY^NTN^ and NT-mock) and mock inoculated NahG-Désirée (NahG-mock) there was no callose accumulating. Callose deposition was observed only at the sites of necrotic lesions in PVY^NTN^-inoculated NahG-Désirée leaves. Two samples of callose accumulation at the site of necrotic lesion are demonstrated (NahG-PVY^NTN^). (Blue for aniline blue, red for background fluorescence, gray for transmission field, most bottom panel for superposition) (PDF 511 kb) Additional file 2:
**Statistical analysis of PVY accumulation and photosynthetic parameters.** The significance of increase (+++: *p <* 0.001, ++: *p <* 0.01, +: *p <* 0.05, •: *p <* 0.1) or decrease (---: *p <* 0.001, --: *p <* 0.01,-: *p <* 0.05, •: *p <* 0.1) in viral accumulation (PVY) (A) and measured parameters of photosynthetic activity (B) is shown for consecutive time points; example 1:0 dpi denotes statistical comparison between viral accumulation at 1 dpi compared to the amount of viral RNA at 0 dpi. All the data is normalized PVY versus mock treated plants. The statistics for both genotypes is shown: Désirée NT, and NahG-Désirée. An empty field denotes no significance. dpi-days post infection, NA-data not available, Pn- net photosynthetic rate, SPAD- chlorophyll content, Fv’/Fm’-actual photochemical efficiency, Fv/Fm-potential photochemical activity, ETR-electron transport rate, Cond- stomatal Conductivity, Transp- transpiration. (DOCX 20 kb) Additional file 3:
**Primary metabolism-related gene expression changes following PVY** ^**NTN**^ **infection in cv. Désirée (NT) and NahG-Désirée (NahG).** Differentially expressed genes in virus *versus* mock treated plants of cv. Désirée and NahG-Désirée at 1, 3, 4, 5 and 7 dpi are shown. Genes are represented for four selected functional groups (MapMan ontology BINs: photosynthesis [1], major carbohydrate metabolism [2], minor CHO metabolism [3] and glycolysis [4]. Only expression values of statistically significantly (FDR *p <* 0.05) differentially expressed genes in at least one time point are shown. Expression values were log_2_ transformed, and a fold-change difference (log2FC) was calculated for PVY^NTN^ *versus* mock. Values are color-coded as red for up-regulation and green for down-regulation. Transcripts were identified using POCI (Potato Oligo Chip Initiative) [64] and stNIB [78] identifiers. (XLSX 149 kb) Additional file 4:
**Validation of microarray results by RT-qPCR.** Microarray results were validated by analyzing eight biologically relevant genes involved in photosynthesis (chlorophyll a-b binding protein: CAB and RuBisCO activase: RA), defense response (β-1,3-glucanase of three classes: Glu-I, Glu-II, Glu-III and pathogenesis-related protein1b: PR-1b), and sugar metabolism (granule bound starch synthase I: GBSSI and CwINV: cell wall invertase) by quantitative real-time PCR. Expression values were log_2_ transformed, and a fold-change difference (log_2_FC) was calculated for PVY^NTN^ *versus* mock in cv. Désirée and NahG-Désirée at 1, 3, 4, 5 and 7 dpi are shown in the table. Statistically significant values (*p <* 0.05) are marked with bold. (DOCX 27 kb) Additional file 5:
**Transcriptional dynamic changes (log**_**2**_ **FC) of genes involved in primary metabolism visualized with MapMan.** Gene expression for cv. Désirée (A) and NahG-Désirée (B) plants infected with PVY^NTN^ are visualized at 1 dpi, 3 dpi, 4 dpi, 5 dpi and 7 dpi. Only statistically significant DE genes (FDR *p <* 0.05) are shown. Results are color-coded: red up-regulation, green down-regulation. (PDF 438 kb) Additional file 6:
**Gas exchange and fluorescence measurement.** Photosynthetic parameters have been measured for both genotypes (NT-Désirée and NahG-Désirée) as described in methods section. Potato leaves were either mock- or virus-treated and analyzed at 0, 0.13, 1, 3, 4, 5, 7, 8 and 11 dpi for each of the measured leaves. Obtained results for following parameters are listed in the table: Pn- net photosynthetic rate, SPAD-chlorophyll content, Fv’/Fm’- actual photochemical efficiency, Fv/Fm- potential photochemical activity, ETR- electron transport rate, Cond- Stomatal Conductivity, Transp- transpiration. Leaf number: three bottom leaves have been inoculated and numbered from 1 to 3 (1-the bottom most inoculated leave, 3-the upper most inoculated leave) (XLSX 39 kb) Additional file 7:
**Visual representation of the analysis that describes the relationship between net photosynthesis, photochemical efficiency and stomatal conductivity.** The analysis for both genotypes is shown as an animation. Nt: Désirée NT, NahG: NahG-Désirée; blue-values for mock inoculated plants, green-values for PVY inoculated plants; red-model predicted value; gradient of colors denotes days post infection (0-11dpi) (e.g. light blue-0 dpi, dark blue-11dpi). (PDF 18640 kb) Additional file 8:
**Results of proteome analysis. Potato leaves from cv. Désirée (NT) and NahG-Désirée (NahG) were either virus-inoculated (PVY) or mock-inoculated (mock) and lamina was collected at 4 dpi.** Leaves from two potato plants were sampled and pooled together to produce three biological replicates per treatment. LC-MS analysis was made in order to quantify protein abundance in the samples 339 identified peptides are shown in the table together with their IDs, descriptions and spectral counts for each sample (sheet’spectral count’). Results of analysis of variance (ANOVA) are shown in (sheet’ANOVA’). g: p-value between genotypes, s: p-value for interaction between treatment (PVY /mock) g*s: p-value for genotype/treatment interaction. (XLSX 97 kb) Additional file 9:
**Gene expression levels for genes corresponding to proteins listed in Fig.** **5.** The table represents expression values of genes, corresponding to proteins listed in Fig. 5. Data for cv. Désirée (NT) and NahG-Désirée (NahG) plants are shown. Potato genome sequencing consortium (PGSC) protein identifiers [80] and ITAG potato annotations were linked to Potato Oligo Chip Initiative (POCI) identifiers [64] for comparisons between protein and gene expression obtained with microarrays. As different POCI sequences can represent the same gene, allelic variants or closely related gene family multiple hits were often retrieved for single proteins in this database. Protein expression is thus linked to multiple results of microarray analysis. On the other hand it is also possible that some of the proteins no corresponding microarray probe have been identified. (XLSX 17 kb) Additional file 10:
**RT-qPCR primers and probes used.** Quantitative real-time PCR assay targets are specified, together with their IDs, primer and probe sequences are shown together with optimal concentrations to be used in the reaction. (DOCX 21 kb)

## Abbreviations

PVY: Potato virus Y
SA: Salicylic acid
dpi: Days post infection
ETR: Electron transport rate
Pn: Net photosynthesis
NT: Nontransgenic plants
RuBisCO: Ribulose 1,5-bisphosphate carboxylase/oxygenase
PSI/PSII: Photosystem I/photosystem II
Fv’/Fm’: Actual photochemical efficiency ratio
