# Erratum to: Bimodal dynamics of primary metabolism-related responses in tolerant potato-*Potato virus Y* interaction

**DOI:** 10.1186/s12864-017-3611-z

**Published:** 2017-03-13

**Authors:** Tjaša Stare, Živa Ramšak, Andrej Blejec, Katja Stare, Neža Turnšek, Wolfram Weckwerth, Stefanie Wienkoop, Dominik Vodnik, Kristina Gruden

**Affiliations:** 1 Department of Biotechnology and Systems Biology, National Institute of Biology, Vecna pot 111, Ljubljana, Slovenia; 2 Department of Ecogenomics and Systems Biology, Faculty of Life Sciences, University of Vienna, Vienna, Austria; 3 Department of Agronomy, Biotechnical Faculty, University of Ljubljana, Ljubljana, Slovenia

## Erratum

While reanalyzing of our recently published data (*Bimodal dynamics of primary metabolism - related responses in tolerant potato-potato virus Y interaction)* [1] for the purpose of detailed transcriptome vs proteome comparison, we realized that an error occurred in annotation of samples in proteomics dataset. The samples collected from NT (nontransgenic) plants were annotated as *NahG* and vice versa. As the proteomic dataset is covering only limited number of proteins, we could not make any biological conclusion based on them. Consequently, the error resulted in a very slight change of the results and does not affect the main results or conclusions of the performed work. The corrections are noted below and the corrected can be found in the attachment.

## Corrected text

Figure legend 1: Please change the value '**-*p <* 0.1' with '**-p < 0.01'

Page 11 and 12: Section Results

Please replace: In the nontransgenic plants, the viral infection resulted in significantly lower abundances of the proteins involved in photorespiration.

*With the amended text: In the NahG-Désirée plants, the viral infection resulted in significantly lower abundances of the proteins involved in photorespiration.*

Please move this sentence to the next paragraph (see the next amendment):

*Activation of Calvin cycle-related transcripts has been detected at 3 days post viral infection and with a time shift of 1 day this effect is reflected also on protein level.*

Please change the paragraph: In the virus-infected NahG-plants, there were lower levels of PSII-associated oxygen-evolving enhancer protein 1 detected. This repression corresponded to the measurements at the level of the transcripts, where the same trend of down-regulation in the expression of the PSII-related genes was observed at 4 dpi (Additional file 3). As in the nontransgenic Désirée plants, the Calvin cycle was also affected in the NahG-Désirée but this time at the point of conversion of glyceralaldehyde-3-phosphate to dihydroxyacetone-3-phosphate with higher abundance of the enzyme triosephosphate isomerase (Fig. 5). In addition to photosynthesis-related proteins, differential abundance of proteins involved in other functions has also been detected (Fig. 5). Virus-dependent induction of histone H2A and CLP protease was shown to work in a SA signaling- dependent manner.

**Fig. 5:**
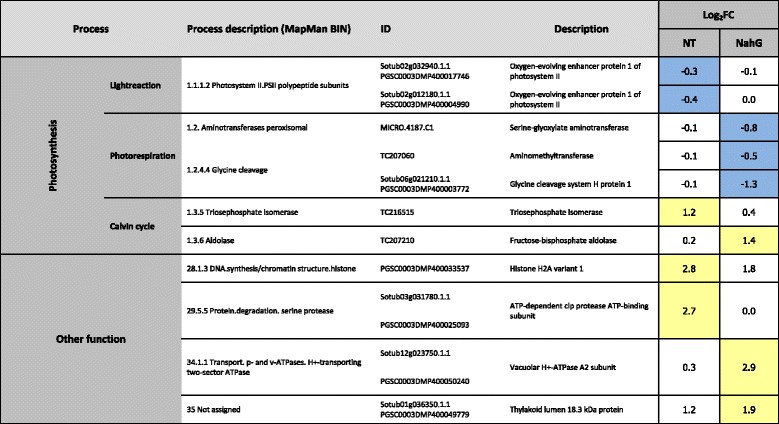
Virus-affected protein abundance. The table shows proteins whose expressions have been up-regulated or down-regulated due to PVY infection. Proteins are grouped according to their function, as determined by MapMan ontology. Samples collected at 4 dpi were analyzed. The average ratios (log_2_FC) of the protein abundance in PVY *versus* mock inoculated plants are shown. Only proteins that showed statistically significantly differentially expression (*p <* 0.05) in at least one genotype are included. Significance is marked with shading; Blue - significantly decreased proteins, yellow - significantly induced proteins. NT – cv. Desiree; NahG – NahG-Désirée]

*With the amended text: In the virus-infected nontransgenic Désirée, there were lower levels of PSII-associated oxygen-evolving enhancer protein 1 detected. As in the NahG-Désirée plants, the Calvin cycle was also affected in the nontransgenic Désirée but this time at the point of conversion of glyceralaldehyde-3-phosphate to dihydroxyacetone-3-phosphate with higher abundance of the enzyme triosephosphate isomerase (Fig.* 5 *). Activation of Calvin cycle-related transcripts has been detected at 3 days post viral infection and with a time shift of 1 day this effect is reflected also on protein level*.

Fig. 5 and Additional file 8 were corrected – 'NT' and 'NahG' results were reversed

## Additional file

Additional file 8: Results of proteome analysis. Potato leaves from cv. Désirée (NT) and NahG-Désirée (NahG) were either virus-inoculated (PVY) or mock-inoculated (mock) and lamina was collected at 4 dpi.] (XLSX 92 kb)
