# Supplementary material for: Bimodal dynamics of primary metabolism-related responses in tolerant potato-Potato virus Y interaction
Source: BMC Genomics. 2015 Sep 19;16(1):716. doi: 10.1186/s12864-015-1925-2 (PMC4575446; doi:10.1186/s12864-015-1925-2)
Supplement: Additional file 2: — Statistical analysis of PVY accumulation and photosynthetic parameters. The significance of increase (+++: p < 0.001, ++: p < 0.01, +: p < 0.05, •: p < 0.1) or decrease (---: p < 0.001, --: p < 0.01,-: p < 0.05, •: p < 0.1) in viral accumulation (PVY) (A) and measured parameters of photosynthetic activity (B) is shown for consecutive time points; example 1:0 dpi denotes statistical comparison between viral accumulation at 1 dpi compared to the amount of viral RNA at 0 dpi. All the data is normalized PVY versus mock treated plants. The statistics for both genotypes is shown: Désirée NT, and NahG-Désirée. An empty field denotes no significance. dpi-days post infection, NA-data not available, Pn- net photosynthetic rate, SPAD- chlorophyll content, Fv’/Fm’-actual photochemical efficiency, Fv/Fm-potential photochemical activity, ETR-electron transport rate, Cond- stomatal Conductivity, Transp- transpiration. (DOCX 20 kb) [file 12864_2015_1925_MOESM2_ESM.docx]

Additional file 7: Statistical analysis of PVY accumulation and photosynthetic parameters.

A) Statistical analysis of viral RNA (PVY) accumulation

|  | dpi | PVY |
| --- | --- | --- |
| Désirée NT | **1:0** | +++ |
|  | **3:1** | -- |
|  | **4:3** |  |
|  | **5:4** | + |
|  | **7:5** | + |
|  |  |  |
| Désirée-NahG | **1:0** | +++ |
|  | **3:1** |  |
|  | **4:3** | ++ |
|  | **5:4** |  |
|  | **7:5** | • |

B) Statistical analysis of photosynthetic parameters

|  | dpi | Pn | SPAD | Fv'/Fm' | Fv/Fm | ETR | Cond | Trans |
| --- | --- | --- | --- | --- | --- | --- | --- | --- |
| Désirée NT | **0.13:0** |  |  |  |  |  |  |  |
|  | **1:0.13** |  |  |  |  |  | + |  |
|  | **3:1** |  |  |  |  |  |  |  |
|  | **4:3** |  |  |  |  | NA | • | - |
|  | **5:4** | --- |  | -- | NA | - | -- | -- |
|  | **7:5** | +++ |  | + |  |  | + | + |
|  | **8:7** |  |  | ++ | - |  |  |  |
|  | **11:8** | -- |  | --- |  |  | - | - |
|  |  |  |  |  |  |  |  |  |
| Désirée- NahG | **0.13:0** |  |  |  |  |  |  |  |
|  | **1:0.13** |  |  |  |  |  |  |  |
|  | **3:1** |  |  |  |  |  |  |  |
|  | **4:3** | + |  |  |  | NA |  | - |
|  | **5:4** | --- | • | - | NA | • | - | • |
|  | **7:5** |  |  |  |  |  | • | + |
|  | **8:7** |  |  | - |  |  |  |  |
|  | **11:8** |  |  |  |  |  |  |  |

The significance of increase (+++: p<0.001, ++: p<0.01, +: p< 0.05, •: p<0.1) or decrease (---: p<0.001, --: p<0.01,-: p< 0.05, •: p<0.1) in viral accumulation (PVY) (A) and measured parameters of photosynthetic activity (B) is shown for consecutive time points; example 1:0dpi denotes statistical comparison between viral accumulation at 1dpi compared to the amount of viral RNA at 0dpi. The statistics for both genotypes is shown: Désirée NT, and Désirée-NahG. An empty field denotes no significance, NA-data not available

Pn- net photosynthetic rate, SPAD- chlorophyll content, Fv'/Fm'-actual photochemical efficiency, Fv/Fm-potential photochemical activity, ETR-electron transport rate, Cond- Stomatal Conductivity, Transp- transpiration.
