# Supplementary material for: Bimodal dynamics of primary metabolism-related responses in tolerant potato-Potato virus Y interaction
Source: BMC Genomics. 2015 Sep 19;16(1):716. doi: 10.1186/s12864-015-1925-2 (PMC4575446; doi:10.1186/s12864-015-1925-2)
Supplement: Additional file 4: — Validation of microarray results by RT-qPCR. Microarray results were validated by analyzing eight biologically relevant genes involved in photosynthesis (chlorophyll a-b binding protein: CAB and RuBisCO activase: RA), defense response (β-1,3-glucanase of three classes: Glu-I, Glu-II, Glu-III and pathogenesis-related protein1b: PR-1b), and sugar metabolism (granule bound starch synthase I: GBSSI and CwINV: cell wall invertase) by quantitative real-time PCR. Expression values were log2 transformed, and a fold-change difference (log2FC) was calculated for PVYNTN versus mock in cv. Désirée and NahG-Désirée at 1, 3, 4, 5 and 7 dpi are shown in the table. Statistically significant values (p < 0.05) are marked with bold. (DOCX 27 kb) [file 12864_2015_1925_MOESM4_ESM.docx]

| **Gene name** | **Target probe**  **(POCI ID)** | **Désirée** | | | | | | | | | | **NahG-Désirée** | | | | | | | | | |
| --- | --- | --- | --- | --- | --- | --- | --- | --- | --- | --- | --- | --- | --- | --- | --- | --- | --- | --- | --- | --- | --- |
|  |  | **1dpi** | | **3dpi** | | **4dpi** | | **5dpi** | | **7dpi** | | **1dpi** | | **3dpi** | | **4dpi** | | **5dpi** | | **7dpi** | |
|  |  | μarray | qPCR | μarray | qPCR | μarray | qPCR | μarray | qPCR | μarray | qPCR | μarray | qPCR | μarray | qPCR | μarray | qPCR | μarray | qPCR | μarray | qPCR |
| **CAB** | cSTD1O21THB | **1.4** | 0.3 | 0.3 | -0.4 | **1.1** | 1.3 | -0.5 | 0.4 | 0.1 | 2.1 | 1.0 | -0.8 | 0.1 | 0.0 | 0.0 | -0.6 | 0.2 | 1.3 | -0.1 | 0.6 |
|  | MICRO.331.C90 | 0.1 |  | 0.0 |  | 0.5 |  | 0.1 |  | -0.1 |  | -0.1 |  | -0.1 |  | 0.0 |  | 0.2 |  | 0.0 |  |
|  | MICRO.331.C84 | 1.2 |  | 0.6 |  | 0.6 |  | -0.6 |  | -0.6 |  | -1.1 |  | -0.5 |  | **-1.4** |  | -0.7 |  | -1.0 |  |
|  | MICRO.331.C81 | **3.5** |  | **2.9** |  | **2.7** |  | **3.3** |  | **3.0** |  | -0.8 |  | -0.5 |  | 0.6 |  | 0.5 |  | -0.8 |  |
|  | MICRO.331.C62 | **0.9** |  | **0.8** |  | **0.8** |  | **0.8** |  | 0.2 |  | **-0.9** |  | 0.1 |  | 0.0 |  | 0.2 |  | -0.3 |  |
|  | MICRO.331.C60 | 0.2 |  | -0.1 |  | **1.8** |  | -0.1 |  | -0.3 |  | -0.4 |  | 0.2 |  | -0.6 |  | 0.5 |  | 0.0 |  |
|  | MICRO.331.C89 | 1.1 |  | -0.4 |  | **2.3** |  | -0.9 |  | 0.2 |  | -1.4 |  | 1.2 |  | 0.3 |  | 1.6 |  | 0.7 |  |
|  | MICRO.331.C79 | 0.6 |  | -0.1 |  | **2.2** |  | -0.5 |  | -0.4 |  | -0.3 |  | -0.2 |  | -1.8 |  | 0.4 |  | 0.1 |  |
|  | MICRO.331.C76 | 0.9 |  | -0.2 |  | **1.4** |  | -0.6 |  | -0.3 |  | 0.7 |  | -0.1 |  | -0.1 |  | 0.3 |  | 0.3 |  |
|  | MICRO.331.C27 | 1.7 |  | 0.3 |  | **2.5** |  | -0.1 |  | 1.3 |  | -1.7 |  | 0.8 |  | 1.0 |  | 1.3 |  | 0.4 |  |
|  | MICRO.331.C12 | **0.8** |  | **0.7** |  | 0.5 |  | 0.1 |  | 0.1 |  | **0.8** |  | 0.0 |  | 0.4 |  | 0.4 |  | -0.4 |  |
|  | MICRO.331.C9 | 0.5 |  | 0.1 |  | **1.8** |  | -0.1 |  | 0.1 |  | 0.3 |  | 0.5 |  | 0.4 |  | 0.8 |  | 0.3 |  |
|  |  |  |  |  |  |  |  |  |  |  |  |  |  |  |  |  |  |  |  |  |  |
| **CwINV** | STMIM75TV | -0.6 | -0.6 | 0.3 | 0.0 | 0.0 | -1.0 | 0.1 | -1.7 | 0.5 | 1.0 | 0.4 | 1.1 | -0.5 | **1.0** | **1.4** | 1.1 | **1.5** | 2.2 | **1.8** | 1.7 |
|  |  |  |  |  |  |  |  |  |  |  |  |  |  |  |  |  |  |  |  |  |  |
| **GBSS1** | MICRO.920.C5 | 0.2 | 0.4 | -0.3 | 0.4 | 0.3 | 0.9 | **-1.4** | -0.8 | -0.3 | 0.3 | -0.5 | -2.1 | 0.0 | -0.3 | -1.3 | **-1.5** | -0.3 | -0.3 | -0.5 | -0.1 |
|  | MICRO.920.C2 | 0.3 |  | -0.4 |  | 0.7 |  | -1.5 |  | 0.0 |  | -0.4 |  | 0.1 |  | -1.4 |  | -0.2 |  | -0.4 |  |
|  |  |  |  |  |  |  |  |  |  |  |  |  |  |  |  |  |  |  |  |  |  |
| **Glu-I** | MICRO.2526.C3 | 0.2 | **1.5** | -0.1 | -0.2 | 0.0 | **1.8** | -0.2 | -0.6 | 0.1 | 1.8 | **0.3** | 4.5 | 0.2 | 0.6 | 0.3 | 2.8 | 0.1 | 2.2 | **0.4** | 3.0 |
|  |  |  |  |  |  |  |  |  |  |  |  |  |  |  |  |  |  |  |  |  |  |
| **Glu-II** | MICRO.2286.C42 | -0.6 | -0.3 | -0.7 | -0.6 | 0.1 | 0.7 | -0.6 | -1.7 | -0.2 | -1.2 | 0.4 | -0.2 | -0.7 | 2.4 | -0.8 | -1.4 | **2.1** | 2.5 | **3.7** | **4.3** |
|  | MICRO.2286.C15 | -0.3 |  | -0.7 |  | 0.2 |  | -0.9 |  | -0.1 |  | 0.7 |  | -0.8 |  | -0.9 |  | **1.8** |  | **3.9** |  |
|  |  |  |  |  |  |  |  |  |  |  |  |  |  |  |  |  |  |  |  |  |  |
| **Glu-III** | MICRO.6187.C2 | -0.4 | -0.5 | -0.3 | -0.5 | 0.4 | 0.5 | 0.2 | -1.0 | 0.4 | -0.4 | -0.3 | -0.2 | -1.1 | -0.5 | 1.4 | 0.5 | **3.6** | **3.1** | **3.9** | 4.6 |
|  | MICRO.6187.C1 | -0.4 |  | -0.2 |  | 0.9 |  | -0.1 |  | 0.4 |  | 0.1 |  | -1.8 |  | 0.6 |  | **3.8** |  | **4.5** |  |
|  |  |  |  |  |  |  |  |  |  |  |  |  |  |  |  |  |  |  |  |  |  |
| **PR-1b** | MICRO.5426.C4 | -0.2 | -0.6 | -1.7 | -0.8 | 0.5 | 1.3 | -1.3 | -1.6 | 1.3 | 1.5 | -1.2 | -3.8 | -2.3 | -2.0 | -0.4 | 0.0 | **4.4** | **5.6** | **5.9** | **5.4** |
|  |  |  |  |  |  |  |  |  |  |  |  |  |  |  |  |  |  |  |  |  |  |
| **RA** | MICRO.4141.C1 | 0.2 | -0.6 | -0.4 | **-1.0** | -0.4 | -0.6 | **-1.3** | **-1.5** | **-1.7** | **-1.8** | 0.6 | -0.9 | -0.3 | -0.4 | -1.0 | **-0.9** | -0.4 | 0.5 | -0.1 | 0.8 |

Additional file 3: Validation of microarray results by RT-qPCR.

.
