# Supplementary figures and images for: Bimodal dynamics of primary metabolism-related responses in tolerant potato-Potato virus Y interaction
Source: BMC Genomics. 2015 Sep 19;16(1):716. doi: 10.1186/s12864-015-1925-2 (PMC4575446; doi:10.1186/s12864-015-1925-2)

## A Transcriptional dynamics Désirée -NT

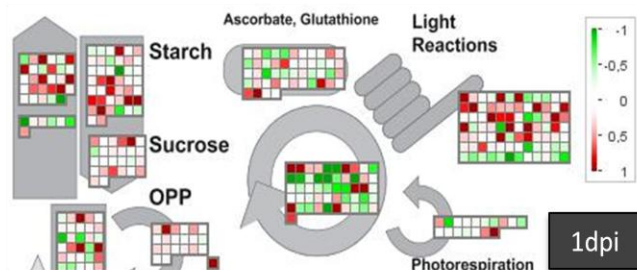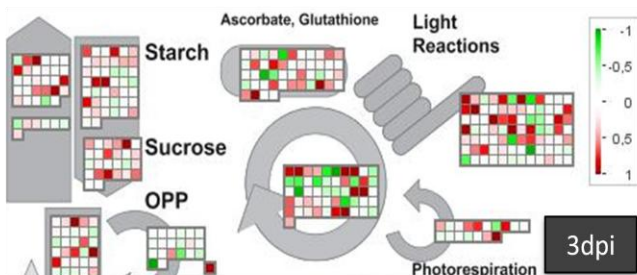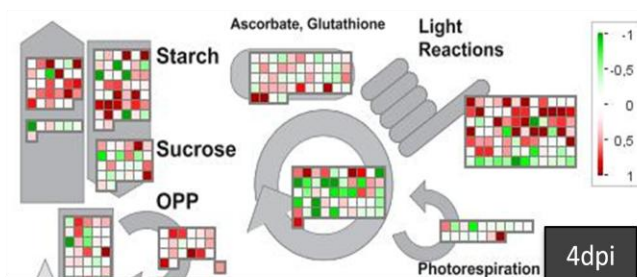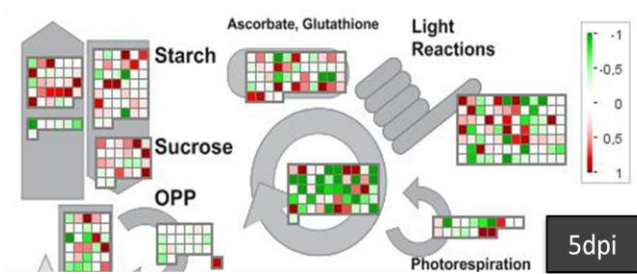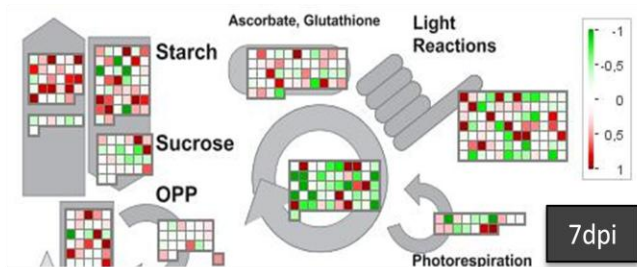

## B Transcriptional dynamics Désirée -NahG

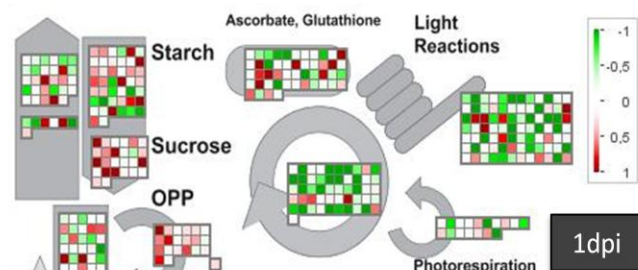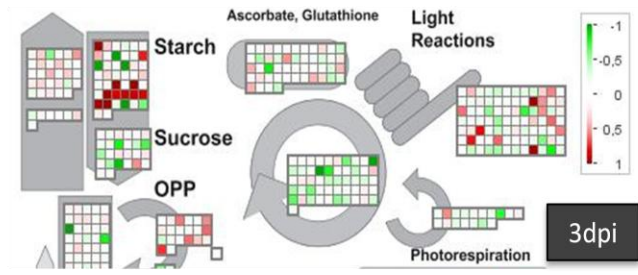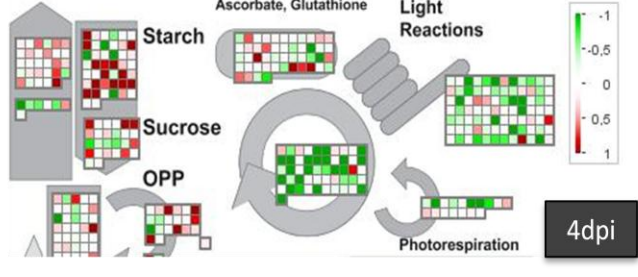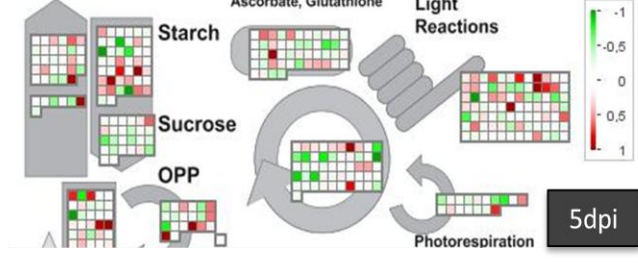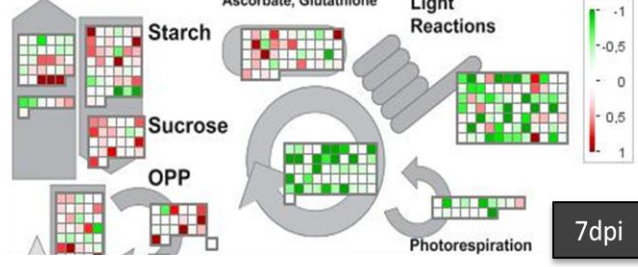

Supplement: Additional file 5: — Transcriptional dynamic changes (log 2 FC) of genes involved in primary metabolism visualized with MapMan. Gene expression for cv. Désirée (A) and NahG-Désirée (B) plants infected with PVYNTN are visualized at 1 dpi, 3 dpi, 4 dpi, 5 dpi and 7 dpi. Only statistically significant DE genes (FDR p < 0.05) are shown. Results are color-coded: red up-regulation, green down-regulation. (PDF 438 kb) [file 12864_2015_1925_MOESM5_ESM.pdf]
