# Supplementary material for: Bimodal dynamics of primary metabolism-related responses in tolerant potato-Potato virus Y interaction
Source: BMC Genomics. 2015 Sep 19;16(1):716. doi: 10.1186/s12864-015-1925-2 (PMC4575446; doi:10.1186/s12864-015-1925-2)

Nt

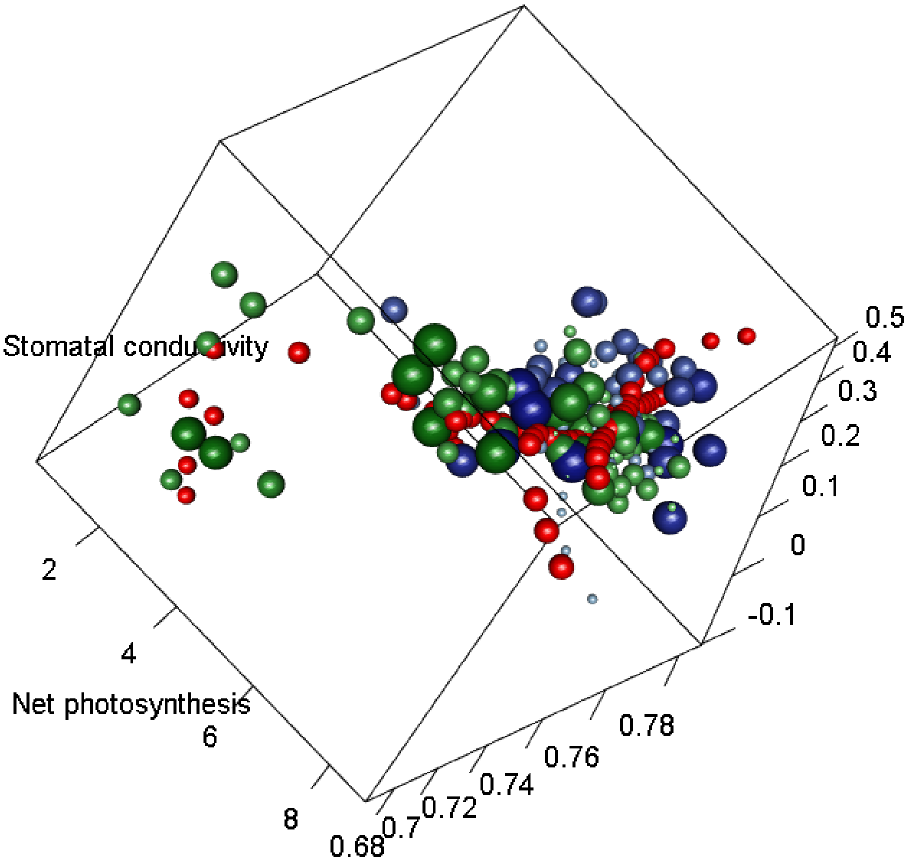

Stomatal conductivity
Net photosynthesis
2
4
6
8
0.68
0.7
0.72
0.74
0.76
0.78
0.5
0.4
0.3
0.2
0.1
0
-0.1

NahG

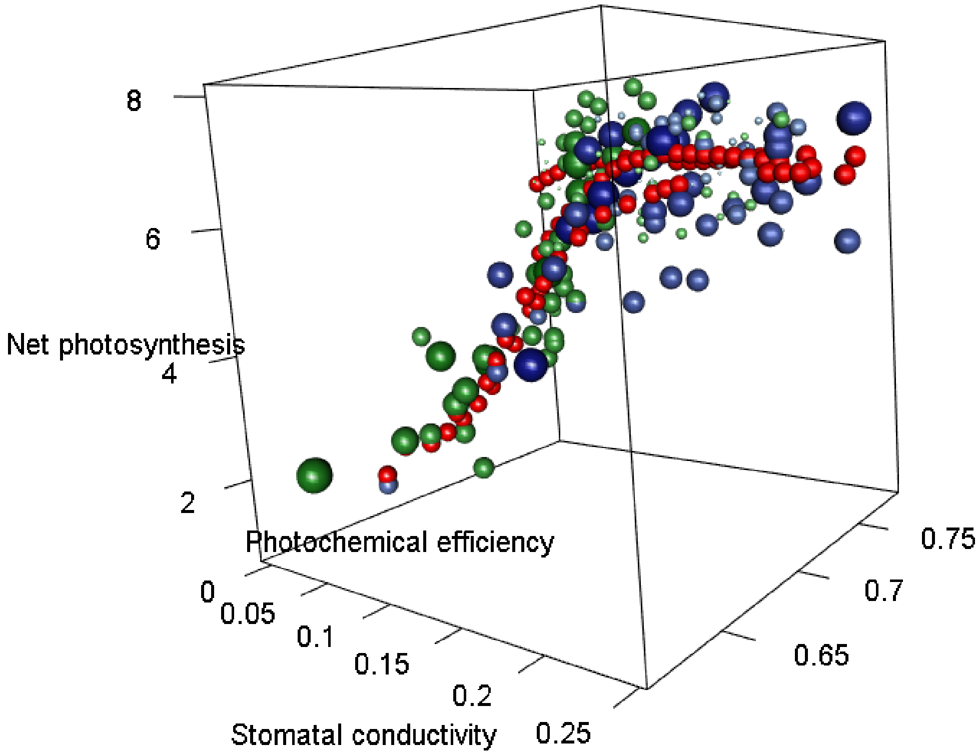

8
6
Net photosynthesis
4
2
0
0.05
0.1
0.15
0.2
0.25
Stomatal conductivity
Photochemical efficiency
0.65
0.7
0.75

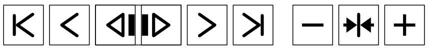

Supplement: Additional file 7: — Visual representation of the analysis that describes the relationship between net photosynthesis, photochemical efficiency and stomatal conductivity. The analysis for both genotypes is shown as an animation. Nt: Désirée NT, NahG: NahG-Désirée; blue-values for mock inoculated plants, green-values for PVY inoculated plants; red-model predicted value; gradient of colors denotes days post infection (0-11dpi) (e.g. light blue-0 dpi, dark blue-11dpi). (PDF 18640 kb) [file 12864_2015_1925_MOESM7_ESM.pdf]
