# Supplementary material for: Bimodal dynamics of primary metabolism-related responses in tolerant potato-Potato virus Y interaction
Source: BMC Genomics. 2015 Sep 19;16(1):716. doi: 10.1186/s12864-015-1925-2 (PMC4575446; doi:10.1186/s12864-015-1925-2)
Supplement: Additional file 10: — RT-qPCR primers and probes used. Quantitative real-time PCR assay targets are specified, together with their IDs, primer and probe sequences are shown together with optimal concentrations to be used in the reaction. (DOCX 21 kb) [file 12864_2015_1925_MOESM10_ESM.docx]

**Additional file 9: RT-qPCR primers and probes used.**

| **Target** | **PGSC target ID** | **Sequence (5'-3')** | **Primer/probe concentration (nM)** | **Amplicon efficiency** |
| --- | --- | --- | --- | --- |
| **PR-1b**  Pathogenesis related protein 1b | PGSC0003DMG400002027  PGSC0003DMG400002028  PGSC0003DMG400002029 | F: GTATGAATAATTCCACGTACCATATGTTC R: GTGGAAACAAGAAGATGCAATACTTAGT | 300 300 | (Baebler et al., 2011) |
| **GBSS1**  Granule-bound starch synthase I | PGSC0003DMG400012111 | F: CCAAGAAATGGGAGACATTGCTATTGGG R: TGATCTTATTGTTGAAACAAGGATAACCAAAGCTC | 300 300 | (Kogovšek et al., 2010) |
| **RA**  RuBisCO activase | PGSC0003DMG400019149 | F: AGAGGCAGCACTCGGAGATGC R: GCAACAGGACGCGCTTTTTTCCC | 300 300 | (Kogovšek et al., 2010) |
| **Glu-I**  Glucan endo-1,3-beta-glucosidase, basic (I) | PGSC0003DMG400014351  PGSC0003DMG400020017  PGSC0003DMG400021848  PGSC0003DMG400040260  PGSC0003DMG400047328 | F: ACGCGAGATGGTGGGTACAG R: TCAGCCCTGTTACTGGCACA | 300 300 | (Oufir et al., 2008) |
| **Glu-II**  Glucan endo-1,3-beta-glucosidase, acidic (II) | PGSC0003DMG401010492  PGSC0003DMG401020492  PGSC0003DMG402010490 | F: GATGCCCTTKTGGATTCWATGTA R: GTATCKGAAAGTGGYTGGCCTT | 300 300 | (Kogovšek et al., 2010) |
| **Glu-III**  Glucan endo-1,3-beta-glucosidase, acidic (III) | PGSC0003DMG40001270 | F: CCCTGGAGTTGTTGTAAATGATAAYG R: ATGCYACATACTCRGCCCTTGAGA | 300 300 | (Kogovšek et al., 2010) |
| **CWInv**  Cell Wall Invertase | PGSC0003DMG402028252 | F: CGCGGAGAGAATCACAATTGA R: TCTCCCAATGTTCTAGTGCAACTTT Probe: FAM-CTAAATGCTTGGAGCATGGCTAATG-TAMRA | 900 900 250 | (Petek et al., 2014) |
| **CAB**  Chlorophyll a/b binding protein | PGSC0003DMG400042498 PGSC0003DMG400016695  PGSC0003DMG400013460 PGSC0003DMG401013418 PGSC0003DMG400013415 PGSC0003DMG400013414  PGSC0003DMG400013413 PGSC0003DMG400013412  PGSC0003DMG400013411  PGSC0003DMG400008299  PGSC0003DMG400008298 | F: TTGGTCCATGCACAAAGCAT  R: ACGGCTCCCATCAACACAA  Probe: FAM-TTGGCCATTTGGGCTTGCCAA- Zen Iowa BlackTM FQ | 900 900 250 | 3.2 |

F-forward primer, R-reverse primer

Baebler, Š., Stare, K., Kovač, M., Blejec, A., Prezelj, N., Stare, T., … Gruden, K. (2011). Dynamics of Responses in Compatible Potato - Potato virus Y Interaction Are Modulated by Salicylic Acid. *PLoS ONE*, *6*(12), e29009. doi:10.1371/journal.pone.0029009

Kogovšek, P., Pompe-Novak, M., Baebler, Š., Rotter, A., Gow, L., Gruden, K., … Ravnikar, M. (2010). Aggressive and mild Potato virus Y isolates trigger different specific responses in susceptible potato plants. *Plant Pathology*, *59*(6), 1121–1132. doi:10.1111/j.1365-3059.2010.02340.x

Oufir, M., Legay, S., Nicot, N., Van Moer, K., Hoffmann, L., Renaut, J., … Evers, D. (2008). Gene expression in potato during cold exposure: Changes in carbohydrate and polyamine metabolisms. *Plant Science*. Retrieved from http://agris.fao.org/agris-search/search.do?recordID=US201301553692

Petek, M., Rotter, A., Kogovšek, P., Baebler, S., Mithöfer, A., & Gruden, K. (2014). Potato virus Y infection hinders potato defence response and renders plants more vulnerable to Colorado potato beetle attack. *Molecular Ecology*, *23*(21), 5378–5391. doi:10.1111/mec.12932

|  |
| --- |
