# Supplementary material for: Bimodal dynamics of primary metabolism-related responses in tolerant potato-Potato virus Y interaction
Source: BMC Genomics. 2015 Sep 19;16(1):716. doi: 10.1186/s12864-015-1925-2 (PMC4575446; doi:10.1186/s12864-015-1925-2)

A **Symptoms development-phenotype**

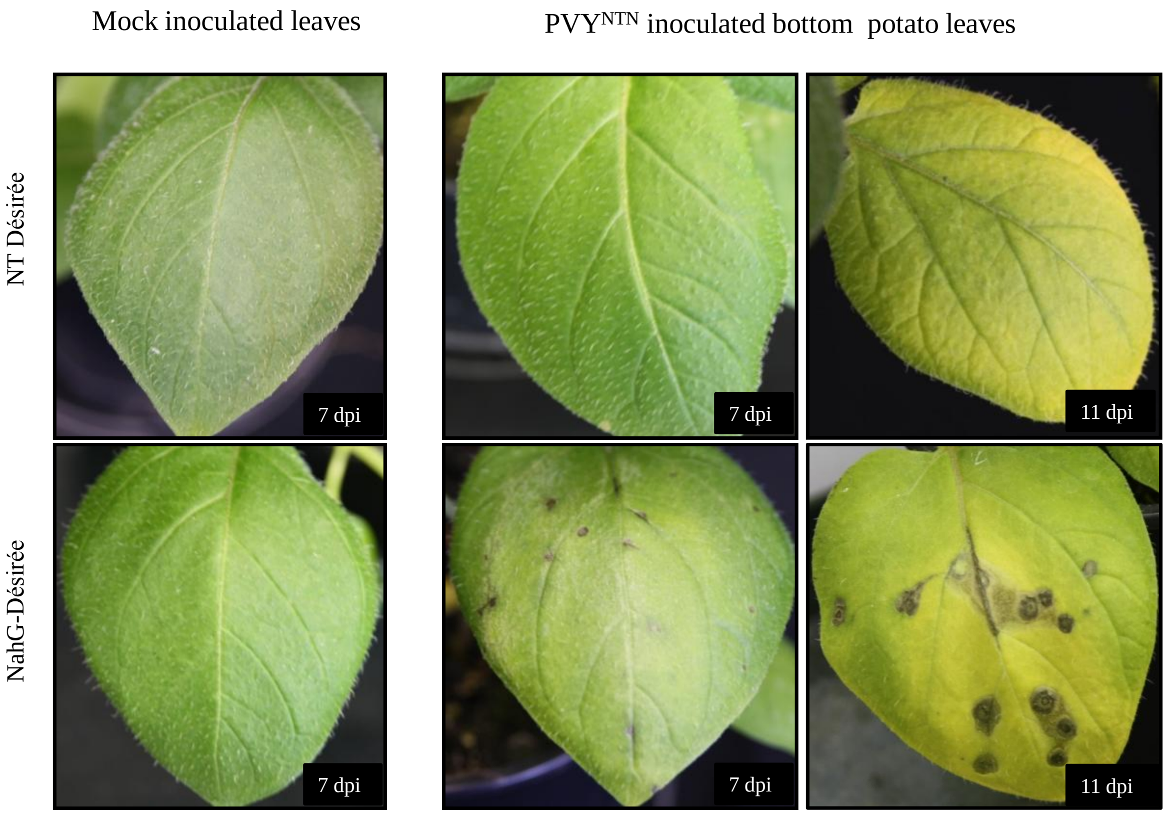

B **Callose staining with aniline blue**

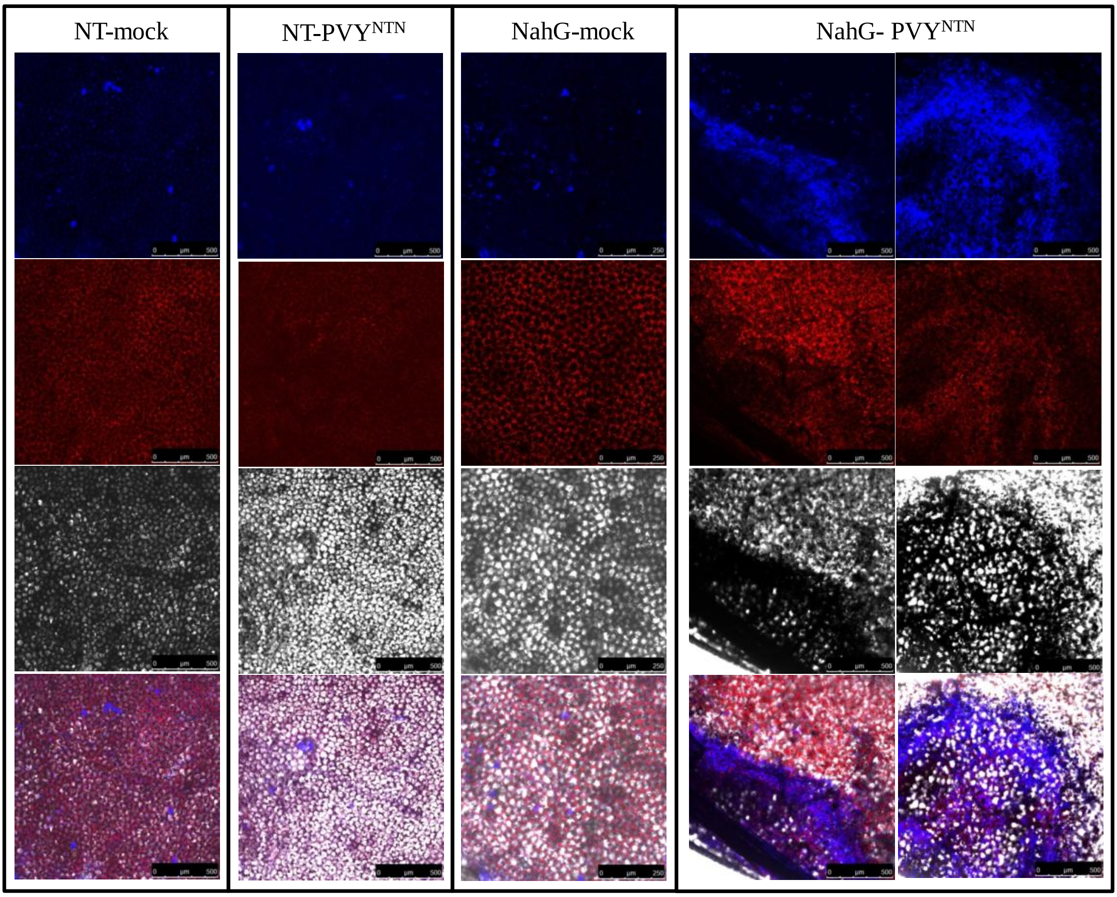

Supplement: Additional file 1: — Symptom development and callose accumulation after PVY NTN inoculation. (A) Phenotypes of the PVYNTN-inoculated (right panel) and mock-inoculated plants (left panel). Symptoms on PVYNTN-inoculated leaves are shown at 7 and 11 dpi, and compared to control mock inoculated leaves for cv. Désirée (upper) and NahG-Désirée (bottom). (B) Callose deposition stained with aniline blue. Leaves of NT Désirée and transgenic NahG-Desiree were either PVY or mock-inoculated. In NT Désirée plants (NT- PVYNTN and NT-mock) and mock inoculated NahG-Désirée (NahG-mock) there was no callose accumulating. Callose deposition was observed only at the sites of necrotic lesions in PVYNTN-inoculated NahG-Désirée leaves. Two samples of callose accumulation at the site of necrotic lesion are demonstrated (NahG-PVYNTN). (Blue for aniline blue, red for background fluorescence, gray for transmission field, most bottom panel for superposition) (PDF 511 kb) [file 12864_2015_1925_MOESM1_ESM.pdf]
